# A spatially explicit empirical model of structural development processes in natural forests based on climate and topography

**DOI:** 10.1111/cobi.13370

**Published:** 2019-08-13

**Authors:** Yuichi Yamaura, David Lindenmayer, Yusuke Yamada, Hao Gong, Toshiya Matsuura, Yasushi Mitsuda, Takashi Masaki

**Affiliations:** ^1^ Department of Forest Vegetation Forestry and Forest Products Research Institute 1 Matsunosato Tsukuba Ibaraki 305‐8687 Japan; ^2^ Fenner School of Environment and Society The Australian National University Canberra ACT 2601 Australia; ^3^ Shikoku Research Center Forestry and Forest Products Research Institute 2–915 Asakuranishi Kochi Kochi 780‐8077 Japan; ^4^ Department of Forest Management Forestry and Forest Products Research Institute 1 Matsunosato Tsukuba Ibaraki 305–8687 Japan; ^5^ Graduate School of Life and Environmental Sciences University of Tsukuba 1‐1‐1 Ten‐noudai Tsukuba Ibaraki 305‐8572 Japan; ^6^ Faculty of Agriculture University of Miyazaki 1‐1 Gakuen Kibanadai Nishi Miyazaki Miyazaki 889–2192 Japan

**Keywords:** broadleaved tree, conifer plantation, DEM, digital elevation model, mapping, old‐growth index, snow depth, temperature, terrain, árbol de hojas anchas, DEM, índice de crecimiento antiguo, mapeo, modelo de elevación digital, plantación de coníferas, profundidad de nieve, temperatura, terreno, 阔叶树, 针叶林林场, 数字高程模型 (DEM), 绘图, 老成林指数, 积雪深度, 温度, 地形

## Abstract

Stand structure develops with stand age. Old‐growth forests with well‐developed stand structure support many species. However, development rates of stand structure likely vary with climate and topography. We modeled structural development of 4 key stand variables and a composite old‐growth index as functions of climatic and topographic covariates. We used a hierarchical Bayesian method for analysis of extensive snap‐shot National Forest Inventory (NFI) data in Japan (n = 9244) to account for differences in stand age. Development rates of structural variables and the old‐growth index exhibited curvilinear responses to environmental covariates. Flat sites were characterized by high rates of structural development. Approximately 150 years were generally required to attain high values (approximately 0.8) of the old‐growth index. However, the predicted age to achieve specific values varied depending on environmental conditions. Spatial predictions highlighted regional variation in potential structural development rates. For example, sometimes there were differences of >100 years among sites, even in the same catchment, in attainment of a medium index value (0.5) after timber harvesting. The NFI data suggested that natural forests, especially old natural forests (>150 years), remain generally on unproductive ridges, steep slopes, or areas with low temperature and deep snow, where many structural variables show slow development rates. We suggest that maintenance and restoration of old natural forests on flat sites should be prioritized for conservation due to the likely rapid development of stand structure, although remaining natural forests on low‐productivity sites are still important and should be protected.

## Abstract 2

*Article impact statement*: Forest structural development rates vary depending on the environment, and in Japan plantations occupy areas where rates of development of stand structure are fast.

## Abstract (es)

Un Modelo Empírico Espacialmente Explícito de Procesos de Desarrollo Estructural en Bosques Naturales Basado en el Clima y la Topografía

**Resumen**

La estructura de un rodal se desarrolla con la edad. Los bosques maduros con una estructura bien desarrollada dan sustento a muchas especies. Sin embargo, las tasas de desarrollo de los rodales probablemente varían con el clima y la topografía. Modelamos el desarrollo estructural de 4 variables clave de un rodal y un índice de crecimiento antiguo compuesto como funciones de covariables climáticas y topográficas. Utilizamos un método bayesiano jerárquico para analizar datos (n = 9,244) en fotografías del inventario nacional forestal (INF) de Japón para entender las diferencias en la edad de los rodales. Las tasas de desarrollo de las variables estructurales y el índice de crecimiento antiguo mostraron respuestas curvilíneas a las covariables ambientales. Los sitios planos se caracterizaron por altas tasas de desarrollo estructural. Por lo general, se requerían 150 años para alcanzar valores altos (∼0.8) en el índice de crecimiento antiguo. Sin embargo, la edad prevista para alcanzar valores específicos varió dependiendo de las condiciones ambientales. Las predicciones espaciales pusieron de relieve la variación regional en las potenciales tasas de desarrollo estructural. Por ejemplo, a veces había diferencia de >100 años entre sitios, aun en la misma cuenca, en el consecución de un valor de índice medio (0.5) después de la cosecha de madera. Los datos de INF sugieren que los bosques naturales, especialmente los bosques maduros (>150 años) permanecen generalmente en crestas improductivas, pendientes pronunciadas o en áreas con baja temperatura y nieve profunda, donde muchas variables estructurales muestran tasas de desarrollo lentas. Sugerimos que el mantenimiento y la restauración de bosques naturales maduros en sitios planos deben priorizarse para conservación debido al probable desarrollo rápido de la estructura del rodal, aunque los bosques naturales restantes en sitios de baja productividad también son importantes y deben ser protegidos.

## 摘要

林分结构随着林龄的增长而逐步发展。有良好林分结构的老成林为许多物种提供了生境。然而, 林分结构的发展速率可能会受到气候和地形的影响。我们以气候和地形为协变量, 对四个关键林分变量的结构发展和一个综合老成林指数构建了函数模型。利用多层贝叶斯模型, 本研究分析了大量日本国家森林资源调查的快照数据 (*n* = 9,244) 以探究林分年龄的差异。结果表明, 结构变量的发展速率及老成林指数对环境协变量表现出曲线响应。地形平坦的地区林分结构发展更快, 需要约 150 年的时间使老成林指数达到较高水平 (∼0.8); 然而, 老成林指数达到特定数值所需的估计时间也因环境条件而异。空间预测的结果突出了可能的结构发展速率在区域间的差异。例如, 森林采伐后, 同一流域的不同位点达到中等水平的老成林指数 (0.5) 所需时间可能相差百年以上。国家森林资源调查的数据还表明, 天然林 (特别是 150 年以上的天然林) 一般位于非生产性的山脊、陡坡或低温且积雪较深的地区, 这些地区许多结构变量发展速率缓慢。尽管在低生产力地区残余的天然林很重要, 应予以保护, 但我们建议应优先保护和恢复位于平地的老成林, 因为它们林分结构发展更快, **【翻译: 胡怡思; 审校: 聂永刚】**

## Introduction

Forests regrow after harvesting at different rates due to the heterogeneous distribution of environmental conditions that influence site productivity (Perry et al. 2008). These include local factors, such as fine‐scale topography (slope position, slope angle, aspect, etc.), soil type, water and nutrient availability, windiness (Curt et al. 2001), and regional‐ to continental‐climatic conditions, such as temperature, precipitation, and snow cover (McKenney & Pedlar 2003). Environmental controls of forest growth are particularly important because environments are globally changing rapidly (Kirilenko & Sedjo 2007).

Stand structure is the horizontal and vertical distribution of forest components, including tree height and diameter, depth and extent of the crown, and presence and abundance of snags and down woody debris (Helms 1998). Stand structure changes with forest growth and typically becomes more complex over time, a process referred to as *stand structural development* (Franklin et al. 2002). Old‐growth forests are characterized by horizontally and vertically diverse structural attributes (Spies 1997; Franklin et al. 2002) and have many environmental values, such as biodiversity conservation, carbon sequestration, and hydrological regulation (Barlow et al. 2016; Lutz et al. 2018; Watson et al. 2018).

Because old‐growth forests are becoming increasingly scarce worldwide (Mackey et al. 2015; Potapov et al. 2017), indicators of structural complexity have been developed to help identify stands with high conservation value (McElhinny et al. 2005; van Galen et al. 2018). One of these indicators is an old‐growth index, which was devised to distinguish old‐growth Douglas fir (*Pseudotsuga menziesii*) stands in western North America based on 4 key structural variables of a stand: mean tree diameter at breast height (dbh), density of large Douglas fir trees (>100 cm dbh), standard deviation (SD) of tree dbh, and tree density (Acker et al. 1998; Zenner 2004; Whitman & Hagan 2007). In old‐growth forests, the first 3 and the fourth variables typically have large and small values, respectively. An advantage of the old‐growth index is its use of common stand variables that can be calculated readily from tree plot data. These structural variables are also likely to distinguish temperate old‐growth forests in other parts of the world (Burrascano et al. 2013).

Close relationships between forest growth and structural development suggest that site productivity influences development rates of forest structure following disturbance (Franklin et al. 2002). Using the old‐growth index, Larson et al. (2008) demonstrated that, after timber harvesting, forest structure develops most rapidly on productive sites. This suggests that there may be substantial spatial heterogeneity in structural development rates. However, because Larson et al. (2008) used measured tree heights as a proxy of site productivity, it remains unclear which and how environmental factors drive spatial heterogeneity in structural development rates.

Local drivers of site productivity (e.g., topography, soil types, soil nutrients, wetness, and local climate) have been measured in intensive field surveys (e.g., Curt et al. 2001). However, topography can be represented by a digital elevation model (DEM). Other local drivers, which are affected by topography (Swanson et al. 1988), can also be represented by a DEM (Moore et al. 1993). For example, topography influences soil formation (Perry et al. 2008) and can be used to predict tree biomass as an alternative of soil properties (de Castilho et al. 2006). Furthermore, regional to continental climates can be incorporated into the models of site productivity (McKenney & Pedlar 2003; Nothdurft et al. 2012). Therefore, it is possible to model structural development based on spatially explicit environmental covariates.

**Figure 1:**
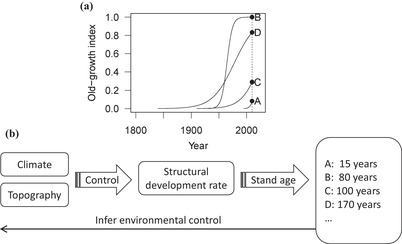
Structural development processes and modeling scheme. (a) Four hypothetical trajectories (A–D) of forest structural development processes that initiated in different years (black circles, states of stand structure surveyed by National Forest Inventory); different forest plots had different stand age (also shown in [b]) and different rates of structural development, which could yield different stand structure even with similar stand age. (b) Structural development rates driven by climate and topography and stand structure driven by stand age and development rates (also shown in [a]). We inferred these latent (unobserved) environmental controls and structural development processes given stand structure, stand age, climate, and topography by assuming they followed the series of formulations in Eqs. 2 and 3.

Our objective was to model structural development processes in natural forests based on climate and topography with the extensive plot data from Japan's National Forest Inventory (NFI) (*n* = 9244 plots). We tested a series of hypotheses (Table 1) to answer the following question: What are the relationships between the development rates of structural variables and climatic and topographic covariates? We used hierarchical Bayesian modeling (Gelman & Hill 2007; Royle & Dorazio 2008), which enabled us to infer underlying structural development processes based on snap‐shot NFI data and to account for differences in stand age among plots (Fig. 1 & Supporting Information). We then applied the model to predict potential structural development rates and values for the old‐growth index across a 20 × 20 km region in central Japan. Maintaining and restoring old‐growth forests may be a key conservation goal (Watson et al. 2018), and we sought to further this objective.

**Table 1:** Possible effects of 6 climatic and topographic covariates on development rates of forest structure

Covariate	Possible effect (reason)^a^	This study^b^	Reference
Climate			
snow depth	negative (as snow depth increases, growth period shortens) unimodal (many tree species have a common optimal climate under which sampled environmental conditions vary widely)	mean dbh: unimodal tree density: positive* large tree density: positive SD of dbh: positive* old‐growth index: positive*	Peterson & Peterson 2001 Nothdurft et al. 2012
warmth index	positive (as the temperature increases, the growth period increases) unimodal (many tree species have a common optimal climate when the sampled environmental conditions vary widely)	mean dbh: negative tree density: negative large tree density: unimodal SD of dbh: unimodal old‐growth index: negative*	McKenney & Pedlar 2003 Nothdurft et al. 2012
Topography			
slope angle	negative (steep slope induces soil movement and tree fall)	mean dbh: negative* tree density: negative* large tree density: positive SD of dbh: neutral old‐growth index: negative	Guariguata 1990
terrain openness (positive openness)	unimodal (concave terrain [small terrain openness] induces soil movement and tree fall; convex terrain [high terrain openness] induces water and nutrient deficiencies; flat terrain has a rapid forest growth rate due to high soil moisture and nutrients)	mean dbh: unimodal tree density: negative large tree density: positive SD of dbh: neutral old‐growth index: neutral	Hunter & Parker 1993; Chen et al. 1998; Curt et al. 2001
catchment area	positive (lower part of slope has rapid forest growth rate due to high soil moisture and nutrients)	mean dbh: positive* tree density: positive* large tree density: positive* SD of dbh: positive* old‐growth index: positive*	Chen et al. 1998; Curt et al. 2001
solar radiation	negative (greater solar radiation induces water deficiency in the temperate zone)	mean dbh: negative* tree density: negative* large‐tree density: positive SD of dbh: negative* old‐growth index: negative*	Chen et al. 1998; Fekedulegn et al. 2003; Mitsuda et al. 2007

a Possible effects and mechanisms are from studies on forest and tree growth (or site productivity).

b Based on the significance of parameter estimates at 5% level. When both simple and quadratic terms were significant (*, indicating nonlinear or curvilinear decrease or increase), the effects of simple terms had higher priority for simplicity. Abbreviation: dbh, diameter breast height.

## Methods

### Plot Data and Usage

Japan's NFI forest monitoring system consists of >13,000 permanent plots on a 4‐km, country‐wide lattice grid. The survey commenced in 1999 and required 5 years to complete a survey of all plots. We obtained stand variables from the plots of natural forests (i.e., we excluded plantations comprising 35% of the data from the analysis). We used all available plot data collected from 1999 to 2013 (4729 plots in which 1934 and 2581 plots were measured 3 and 2 times, respectively) (see Supporting Information for the treatment of NFI data). Tree species composition of Japan's natural forests is influenced by a thermal gradient, specifically a warmth index (corresponding to the period of plant growth) calculated by summing monthly mean temperatures >5°C (Kira 1991). We used only those NFI data within 45–180 for the warmth index to focus on the deciduous and evergreen broad‐leaved forests (cool‐ and warm‐temperate forests [Kira 1991]), which are 2 major forest types in Japan. We excluded subarctic and subtropical forests.

Based on the successful application of an old‐growth index in the United States (e.g., Acker et al. 1998; Zenner 2004; Whitman & Hagan 2007) and the relationships between structural variables and stand age (derived from forest registers) in our NFI data, we used the following 4 structural variables to distinguish Japanese old‐growth forests from younger stands: mean dbh of live trees with >5 cm dbh, density of live trees with 40 cm dbh (large tree density/ha), SD of dbh for live trees with >5 cm dbh, and density of live trees with >5 cm dbh (Supporting Information). The first 3 and the fourth variables typically increase and decrease with stand age, respectively. The correlation coefficients between the 4 structural variables were |0.31|–|0.73| (Supporting Information).

### Climate and Topography Covariates

We used maximum snow depth and the warmth index as climate covariates in our model, both of which are known to affect tree species composition across Japan (Kira 1991; Nakashizuka & Iida 1995), possibly because of their effects on tree survival and regeneration processes (Supporting Information). We used Climate Mesh Data 2000 published by Japan's Meteorological Agency (1‐km resolution) to generate values for these 2 covariates (Supporting Information). We obtained data on topographical covariates (slope angle, terrain [positive] openness, catchment area [surrogate for soil moisture and nutrients] (Table 1), and solar radiation [Supporting Information]), which are known to influence forest growth and structure (e.g., Guariguata 1990; Mitsuda et al. 2007), from a 20‐m DEM based on a 10‐m DEM published by the Geospatial Information Authority of Japan (Supporting Information). We used ArcGIS 10.3 Spatial Analyst extension (ESRI, Redlands, California, U.S.A.) and SAGA GIS (Conrad et al. 2015) for geospatial analyses.

### Model Structure

Following the earlier definition of the old‐growth index, we formulated the old‐growth index (*I*_og_) to have values from 0 to 1:

(1) $$I_{\mathrm{og}}=\mathrm{mean}\left(I_{i,\mathrm{og}}\right)=\frac{1}{4}\times \sum_{i=1}^{4}\left|\frac{x_{i}-x_{i,\mathrm{young}}}{x_{i,\mathrm{old}}-x_{i,\mathrm{young}}}\right|,$$

where *x_i_* is the *i*th structural variable, *x_i,_*_young_ is the median value of *x_i_* for young forests derived from the plot data, and *x_i,_*_old_ is the median value of *x_i_* for qualified old‐growth forests in Japan. This formulation means *I*_og_ is a mean value of 4 subindices calculated from 4 structural variables (*I_i_*_,og_). We used median values rather than mean values, as employed by Acker et al. (1998), to reduce the effects of outliers. Although Larson et al. (2008) obtained *x_i_*_,young_ from 40‐ to 80‐year‐old forests, the stand ages of interest in this study included forests <40 years old because Japan is dominated by young forests (Yamaura et al. 2012). We then set *x_i_*_,young_ by using the plot data from ≤10‐ to 30‐year‐old forests (Supporting Information). To obtain *x_i,_*_old_, we used median values from 18 permanent plots registered as old‐growth deciduous and evergreen broad‐leaved forests (>150 years old) in Japan (Ishihara et al. 2010). This simplification enabled us to analyze the national data set with a single criterion (median value) for each structural variable. We did not find clear differences in structural variables between deciduous and evergreen broad‐leaved forests except for live tree density (Supporting Information). Live tree density also varied little between young and old growth forests; however, we incorporated this variable into the analysis because its development rates showed a dependency on topographic covariates. Nevertheless, modeled environmental dependency for the rates of development of old‐growth index remained mainly unchanged when live tree density was excluded (Supporting Information). We assigned *x_i,_*_young_ or *x_i,_*_old_ for the values of *x_i_* beyond the range of *x_i,_*_young_ and *x_i,_*_old_, and delimited *I_i_*_,og_ within 0 and 1. Although live tree density decreases with stand age, older stands can have higher indices due to the use of absolute values in Eq. 1.

We then examined the effects of environmental covariates on development rates of *I*_og_ and the 4 subindices (*I_i_*_,og_). Following Larson et al. (2008) and Warton and Hui (2011), we logit transformed these proportional data (*I*_og_ and *I_i,_*_og_) and regressed stand age (explanatory variables) on logit‐transformed proportions (as response variables):

(2) $$\begin{array}{ccc} & & \log\left(I_{\mathrm{og},j}/\left[1-I_{\mathrm{og},j}\right]\right)=\beta_{0}+\mathrm{coef}f_{j}\times \mathrm{ag}e_{j}\\ & & \;+\;\mathrm{plot}.\mathrm{ef}f_{\mathrm{site}\left[j\right]}+\mathrm{period}.\mathrm{ef}f_{\mathrm{term}\left[j\right]}+e_{j}, \end{array}$$

where *I*_og,_ *_j_* is the old‐growth index of the *j*th sample (plot data), age*_j_* is its stand age, *β*_0_ is an intercept, coeff*_j_* is a coefficient of stand age (can be specific to the *j*th sample) that dictates structural development rates (Larson et al. 2008), plot.eff is a random plot effect because the same plots could be measured multiple times, and period.eff is a fixed‐effect term that captures the possible effects of measurement period by first and second periods relative to the latest third period (third period was treated as a reference category). As the initial phase of model development for the old‐growth index, we made the intercept constant across the plots to simplify the model following Larson et al. (2008) (but see Supporting Information). Subscripts site (*j*) and term (*j*) denote the identity of the plots and measurement periods for *j*th sample. The final term (*e_j_*) is an unexplained error term with a normal distribution. A challenge in this method is the inability to deal with possible maximum and minimum proportional values (1 and 0) because the denominator of the logit transformation cannot take a value of 0 (*I*_og_ = 1) and its logarithm cannot take 0 (*I*_og_ = 0). We therefore added to, or subtracted from, the minimum nonzero values, called *ε* (Supporting Information), the possible minimum or maximum values (0 or 1 values in proportion) that allowed the application of logit‐transformed regression (Warton & Hui 2011).

We examined effects of environmental covariates on structural development rates by modeling coeff*_j_* as the function of covariates:

(3) $$\mathrm{coef}f_{j}=\beta_{1}+\sum_{k}\left(\beta_{k,1}x_{k,j}+\beta_{k,2}x_{k,j}^{2}\right),$$

where *β*_1_ is an intercept, *x_k,j_* is the *k*th covariate of the *j*th site, and *β_k_*_,1_ and *β_k_*_,2_ are coefficients of *x_k,j_* and its squared term, respectively. Using a quadratic model, we considered the possible nonlinear effects of covariates. Although we also constructed low‐rank thin‐plate splines (one of the generalized linear models) that can deal with more complex functional forms, modeled effects were mostly blurred and not significant (Supporting Information). We therefore used this relatively simple quadratic model (cubic models also yielded the similar results with quadratic models). Our model was hierarchical in that the slope for stand age (coeff*_j_*) in Eq. 2 was a function of covariates in Eq. 3 (varying‐slope model [Gelman & Hill 2007]).

Our approach allowed the use of snap‐shot plot data from stands of different age (Fig. 1). Specifically, each stand can have different structural development rates (coeff*_j_*), depending on the values of environmental covariates (Eq. 3), and their development after disturbance follows logistic curves (Eq. 2). Current development (*I*_og_) is therefore the product of these processes with specific period (stand age). In other words, given current stand and environmental data and the assumed model formulations, we inferred the latent underlying development processes and their possible environmental dependencies (parameters of Eqs. 2 & 3).

Because our NFI data set had 2 consecutive pairs of the structural variables at the same plots, we compared the 4 variables between third and second periods and second and first periods. It was shown that more recent data sets had generally larger values (Supporting Information), which was inconsistent with the expected pattern for live tree density (typically decreases with stand age). This may be due to underestimated tree densities in the older periods of field surveys (Forestry Agency 2017*b*). We therefore incorporated the effects of measurement period in Eq. 2 (period.eff). As an independent data set, we also examined the longitudinal permanent plot data from Ishihara et al. (2010). We derived structural variables from 7 plots of broad‐leaved forests <100 years old with 5–13 years of longitudinal surveys (mean [SD] = 9.9 years [2.5]). The temporal changes of structural variables generally exhibited the expected pattern (Supporting Information). Although we did not include these permanent data in our analysis, their inclusion did not change the obtained results due to their small sample size. In our model, although we simplified variable trajectories of structural development processes depending on a range of factors, such as disturbance history (Spies 1997), we could flexibly alter the model structure. For example, we could consider variability in initial conditions in the intercept of Eq. 2, decadal climatic variability in Eq. 3, or both.

### Model Fitting

To estimate relationships between covariates and structural development rates, we fitted the models (Eqs. 2 & 3) for the old‐growth index (*I*_og_) and for 4 subindices (*I_i,_*_og_). We standardized each covariate to enhance convergence (Kéry & Royle 2016) and stabilized estimates, especially quadratic terms (Schielzeth 2010). Although there was limited correlation between most covariates (|*r*| <0.3), snow depth and warmth index (−0.57) and positive openness and catchment area (−0.62) were highly correlated (Supporting Information). Highly correlated covariates are not always detrimental to an analysis and can be required to explain variations in response variables (e.g., Hamilton 1987). We therefore fitted these correlated covariates simultaneously because we considered that they likely had different ecological meanings and our sample size was large. We also confirmed that parameter estimates remained qualitatively unchanged when we excluded one of the pairs of covariates from the model.

We estimated parameters with Markov Chain Monte Carlo analysis with JAGS version 4.2.0 (Plummer 2013), jagsUI version 1.3.7 (Kellner 2015), and R version 3.2.3 (R Core Team 2015) software. We used conventional vague priors, ran 3 chains with different initial values, discarded the initial 100 iterations, and ran an additional 10,000 iterations to examine the posterior distributions (change of the prior distributions did not affect the estimates [Supporting Information]). Chain convergence was achieved when the $\bar{R}$ statistic was <1.1 for parameter estimates; otherwise, an additional 10,000 iterations were conducted until convergence in the autojags function in jagsUI. We calculated the coefficient of determination (*R* ^2^) from the Pearson correlation coefficient between observed and predicted values at logit scale. To calculate *R* ^2^, we excluded random plot effects but included measurement period effects in Eq. 2. We also tested the model performance by cross‐validation (holdout method). We randomly chose 90% of the data for model training and tested the model with the 10% of remaining data. We repeated this procedure 100 times and obtained *R* ^2^ at each repetition. We did not undertake model selection because many covariates had significant effects (their 95% CI did not include zero) such that we could compare the fitted functional forms among the structural variables given the large sample size (Supporting Information).

### Mapping Indices

To demonstrate the utility of our models, we made spatial predictions of the old‐growth index for natural forests in northern Ibaraki Prefecture, central Japan (between the cities of Kitaibaraki and Takahagi). We compiled relevant climate, topographic, and stand (forest type and stand age) covariates based on the above‐described methods for every 20‐m resolution grid. We derived stand covariates from forest registers.

Because our model for the old‐growth index treated structural development processes and stand age as functions of climate and topography, we could estimate the stand age required to attain a certain value of the old‐growth index under specific environmental conditions. Following Larson et al. (2008), we inferred these ages at values of 0.5 old‐growth index (*t*_0.5_) for every 20‐m grid.

We constructed the model of the old‐growth index substituting parameter estimates (posterior means) and environmental covariates for each grid and equated the linear predictor (right‐hand side of Eq. 2 without random plot and measurement period effects) with 0 (meaning 0.5 value of the index). We then numerically solved this equation for stand age (i.e., obtained corresponding stand age [*t*_0.5_]) with the uniroot function in R. To prevent model extrapolation, we replaced the environmental covariates beyond 95% of the data used for model construction by 97.5% or 2.5% quantile values in predictions.

We also produced a map of predicted values (given the current stand age) of old‐growth index for natural forests. Mean and median posterior estimates were almost the same, and *t*_0.5_ predicted by mean and median estimates was also almost the same and was highly correlated (*r* > 0.99). Finally, we compared the environmental covariates among old natural forests (>150 years old), other natural forests, and major plantation forests in NFI data. We only used the latest third‐period data and did not screen plots by the warmth index.

## Results

### Modeling Structural Development Processes

**Figure 2:**
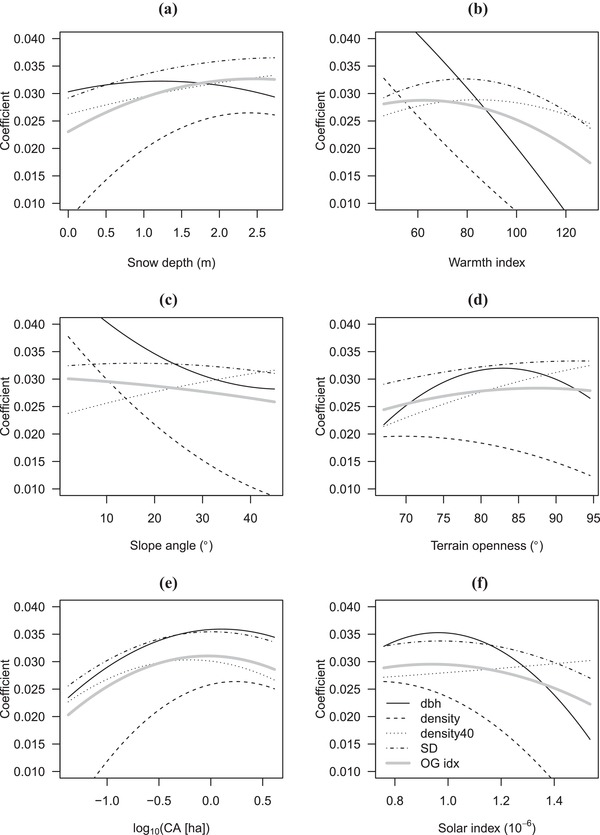
Modeled dependency of 4 forest structural variables and old‐growth index on 6 environmental covariates (dbh, mean diameter at breast height for live trees; density, density of live trees; density 40, density of live trees with 40 cm dbh; SD, standard deviation of dbh for live trees; OG idx, old‐growth index; CA, catchment area). Fitted lines are derived from Eq. 3 and shown in the range of 95% percentiles of environmental covariates. Other covariates were held constant at mean values. See Supporting Information for their corresponding 95% CIs.

Our 4 structural variables, as well as the old‐growth index, exhibited varied and curvilinear responses in their development rates to 6 climatic and topographic covariates (Fig. 2). Mean dbh and tree density were the most sensitive and SD of dbh showed similar response, but its environmental dependency was not as strong. The density of large trees was also not sensitive but showed unique responses, and the old‐growth index had an intermediate response to the 4 structural variables. Specifically, tree density showed clear positive responses to snow depth. Tree density and mean dbh exhibited negative responses to the warmth index. Responses of mean dbh to snow depth and large‐tree density and SD of dbh to the warmth index were unimodal. Structural variables (except for large‐tree density) had consistent negative responses to slope angle and solar radiation, and large‐tree density showed only positive responses to these covariates. Structural variables exhibited varied responses to terrain openness. All structural variables consistently showed positive responses to catchment area.

**Figure 3:**
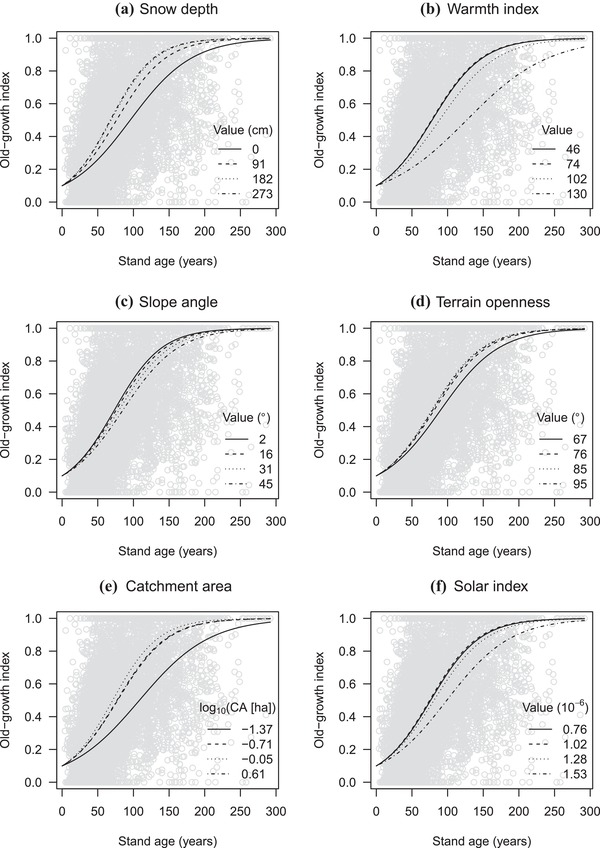
(a–f) Inferred logistic curves of old‐growth index relative to stand age for 6 environmental covariates (CA, catchment area). Curves with different covariate values (within the 95% percentiles) are depicted. For example, (b) shows 4 fitted lines with 4 different values of warmth index (0.025 and 0.975 quantiles and their intermediate values). Other covariates were held constant at mean values. Effects of measurement period are not included (assuming they are from the third period).

Inferred logistic curves showed large unexplained variation in measured structural variables and the old‐growth index (Fig. 3 & Supporting Information). The *R* ^2^ ranged from 0.23 (mean dbh) to 0.31 (SD of dbh), and cross validation yielded 0.02‐0.03 SD of *R* ^2^ around these values (Supporting Information). For example, many plots approximately 50 years old (most common stand age) had higher values for the old‐growth index than predicted by the model (Fig. 3 & Supporting Information). Our results suggested that 150 years was generally required for natural forests to attain high values (approximately 0.8) for the old‐growth index; the specific age to achieve such values varied depending on plot‐level environmental conditions. Snow depth, warmth index, catchment area, and solar radiation had large effects on the forms of logistic curves (Fig. 3). We also found significant measurement‐period effects. While accounting for stand‐age differences given the model structure, the first and second periods of plot measurements produced smaller values of 4 structural variables and old‐growth index compared with the third period (Supporting Information). These deviations were generally larger in the older first period than in the second period.

### Model Prediction

**Figure 4:**
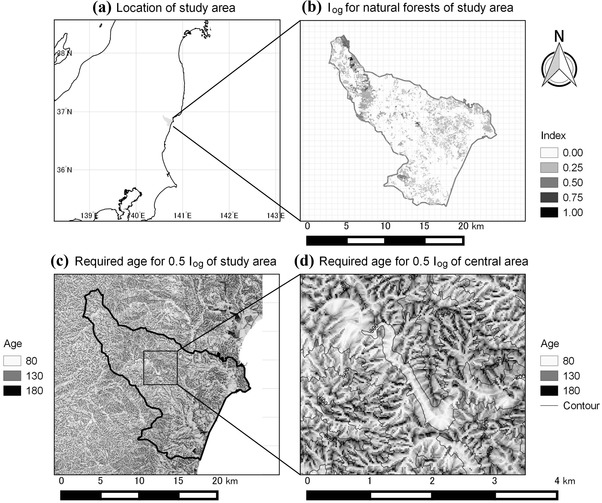
Spatial prediction of structural development processes based on the series of parameter estimates (Eqs. 2 & 3) and associated environmental covariates: (a) location of study area, (b) predicted old‐growth index (I_og_) for natural forests in the study area, (c) predicted stand age required to achieve a 0.5 I_og_ (t_0.5_) in northern Ibaraki prefecture, and (d) predicted stand age required to achieve a 0.5 I_og_ (t_0.5_) incenter of study area. Estimates of I_og_ are available only for natural forests within the middle of the study area (b), where forest‐register data are available. See Supporting Information for the spatial distribution of environmental covariates.

Predicted stand age to achieve a 0.5 value for the old‐growth index (*t*_0.5_) showed spatial heterogeneity in structural development rates associated with climate and topography (Figs. 4c & 4d). Eastern grids had higher *t*_0.5_ (low development rates) due to higher temperatures and limited snow (Fig. 4c). In each catchment, flat valley bottoms had low *t*_0.5_, whereas ridges and steep slopes had high *t*_0.5_ (Figs. 4c & 4d). Large values of *t*_0.5_ were spatially clustered in some areas due to complex terrain (Fig. 4c & Supporting Information). Predicted values of the old‐growth index exhibited substantial spatial heterogeneity (Fig. 4b), and high values were found in the west, where old natural forests occur (Fig. 4b & Supporting Information).

### Environmental Condition Among Forest Types

**Figure 5:**
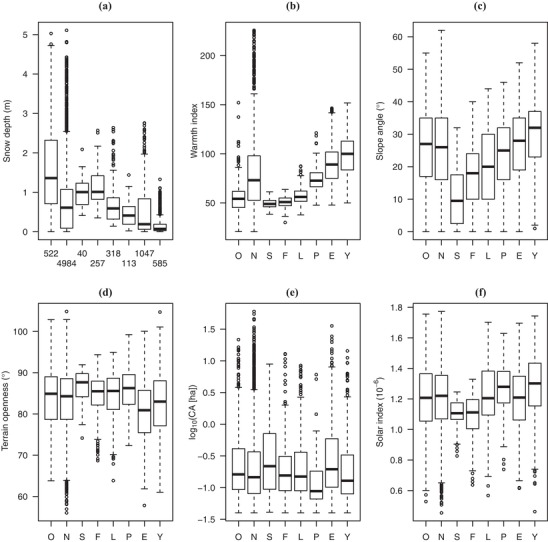
(a–f) Relationship between 6 environmental covariates and 8 forest types covered by National Forest Inventory data (O, natural forest >150 years old; N, natural forest ≤150 years old; S, Sakhalin spruce [Picea glehnii] plantation; F, Sakhalin fir [Abies sachalinensis] plantation; L, Japanese larch [Larix kaempferi] plantation; P, Japanese red pine [Pinus densiflora] plantation; E, Japanese cedar plantation; Y, Japanese cypress [Chamaecyparis obtuse] plantation; CA, catchment area; boxes, 25th and 75th percentiles [interquartile range]; whiskers, minimum and maximum values within 1.5 times interquartile range from interquartile range; horizontal line within bars, median; circles, outliers). In (a), numbers along the horizontal axis indicate the number of plots for the respective forest types shown on the x‐axes of the other graphs.

The NFI data showed that site conditions differed between natural and plantation forests in Japan. Environments supporting natural forests, especially those for old forests, were typically in the areas with deep snow, low temperatures, and large slope angle (Fig. 5). Japanese cedar and cypress plantations, which are 2 primary species of plantations in Japan, have been established in the warmer parts of Japan; cedar plantations dominated the areas with large catchment areas at which development rates of natural forests are high. Japanese red pine plantations were an exception; they have been established in locations with small catchment area.

## Discussion

To the best of our knowledge, we are the first to spatially evaluate structural development processes of natural forests at a regional scale. Our results suggest that old natural forests remain on unproductive areas, where many structural variables have slow rates of development. Our findings also highlighted the conservation importance of old natural forests on flat areas. Given the current scarcity of old‐growth forests and the heterogeneous distribution of rates of structural development, our empirical modeling is useful for assessing the impacts of current land use from the perspective of biodiversity conservation.

### Structural Development Processes and Environment

Mean and SD of dbh and large‐tree density showed unimodal responses to snow depth and the warmth index. Unimodal responses are consistent with the growth rates of a range of tree species in relation to precipitation and temperature from southwest Germany (Nothdurft et al. 2012). There may be common optimal climate conditions for these structural variables. Low structural development rates in areas characterized by deep snow and low temperatures are most likely explained by a short growth period (Peterson & Peterson 2001). Clear positive and negative responses by tree density to snow depth and warmth index, respectively, were partially because evergreen broad‐leaved old‐growth forests in southwestern Japan had high tree densities (Supporting Information).

However, low structural development rates for other variables in areas with limited snow and higher temperatures may not be explained by declines in growth rates but rather by long‐term and intensive forest use by humans. Evergreen broad‐leaved forests have 2 times larger annual net primary production as deciduous broad‐leaved forests, which dominate northeastern Japan (Kira 1991). Japan's southern forests, where warm temperatures and limited snow allow people to use forests throughout the year, have been exploited heavily (Totman 1989). This is consistent with the evidence of there being few old, natural forests (Fig. 5) and natural forests (Yamaura et al. 2011) in those areas in Japan.

Sites with steep slopes or concave terrain were likely to have low development rates for many structural variables. This is most likely due to high soil movement and elevated rates of tree fall (Guariguata 1990). It is also well known that sites with small catchment areas and exposed (convex) topography, such as ridges, have impaired forest growth rates (Curt et al. 2001). An unexpected result was that large‐tree density increased with slope angle and terrain openness. The inaccessibility of steep sites and ridges may lead to reduced human disturbance and in turn more large old trees (Pederson 2010). Solar radiation intensity had mostly negative effects, which was consistent with studies on forest growth rates (Chen et al. 1998) and suggests that water deficiency can also affect structural development rates.

### Old‐Growth Index as an Indicator of Forest Biodiversity

Forest structure is a well‐known indicator of forest biodiversity (Lindenmayer et al. 2000), and the old‐growth index may be a potentially useful composite indicator. Identifying forests with developed structure and high conservation value was a key motivation for devising the old‐growth index (Spies & Franklin 1988, 1991; Whitman & Hagan 2007). Because our model treated the index as a function of stand age, it is straightforward to forecast the future status of old growth in landscapes subject to different kinds of forest management. The old‐growth index comprises common stand variables, and our environmental covariates were also derived from a DEM and a GIS. Our framework is therefore broadly applicable to other regions.

Our results showed that fine‐grained (20‐m resolution) topographic factors can lead to differences in structural development rates (Figs. 4c & 4d). This scale intersects well with the scale of forest management ownership and decision making. For example, 74% of Japanese forest owners have holdings of 1–5 ha (Forestry Agency 2017*c*). In our mapping area, the mean [SD] size of the stand area of natural forests was 0.73 [2.38] ha. It has been suggested that fine‐scale topography provides the underlying setting for ecosystem processes and human land uses (Swanson et al. 1988). It was predicted that topographic features throughout a landscape could lead to differences in >100 years to attain an old‐growth index of 0.5 after harvesting, even within the same catchment (Fig. 4d). We suggest the consideration of topography would be useful for the spatial evaluation of biodiversity and ecosystem services and would go beyond, for example, land use and cover as simple proxies for them (Rieb et al. 2017).

### Temporal Changes in Measurement Error

Our results showed that older surveys that have measured structural variables could yield smaller values. Surveyors can miss trees at the margins of circular plots or measure dbh of tree trunks at a height taller than the prescribed height, which is especially likely for complex‐shaped broad‐leaved trees (Forestry Agency 2017*a*). Underestimation of the SD can be the products of these 2 processes. Accuracy of measurement has been improving since surveyors completed field training from 2011 (Forestry Agency 2017*a*, 2017*b*). Therefore, there is a risk of overestimating development rates without considering these measurement errors.

### Limitations and Implications

Although we simplified our model to allow for scaling up and did not analyze tree species composition and coarse woody debris, these compositional and structural forest attributes characterize different forest development stages and have important functions, such as habitat provision (e.g., Franklin et al. 2002). Relatively large amounts of variation remained unexplained by our model, indicating the potential for model improvement (e.g., with other local information, such as disturbance history and soil type; incorporating environmental covariates into the intercept; and direct modeling of annual changes in structural variables from repeated surveys) (Supporting Information).

Our results suggest that the replacement of natural forests by plantation forests is not a spatially random process. Natural forests, especially old natural forests, remain generally on unproductive ridges, steep slopes, or areas with severe climatic conditions (Fig. 5), where many key attributes of stand structure develop slowly (Fig. 2). Therefore, the impacts of replacement of natural forests by plantations are larger than those indicated by the spatial extent of converted areas alone. The maintenance and restoration of old‐growth forest on flat sites should be prioritized for conservation, although natural forests on unproductive sites will continue to be important and should be protected.

## Supporting information

Descriptions about NFI data (Appendix S1), treatment of structural variables and model structure (Appendix S2), graphical plots of structural variables and varied models (Appendix S3), environmental covariates (Appendix S4), graphical plots of model outputs (Appendix S5), results of cross‐validation (Appendix S6), environmental covariates for spatial prediction (Appendix S7), modeling issue of Eq. 2 (Appendix S8), parameter estimates (Appendix S9), and source R codes of the analysis (Appendix S10) are available online. The authors are solely responsible for the content and functionality of these materials. Queries (other than absence of the material) should be directed to the corresponding author.
