# Supplementary material for: A spatially explicit empirical model of structural development processes in natural forests based on climate and topography
Source: Conserv Biol. 2019 Aug 13;34(1):194–206. doi: 10.1111/cobi.13370 (PMC7027480; doi:10.1111/cobi.13370)
Supplement: Supplementary file 1 — Descriptions about NFI data (Appendix S1), treatment of structural variables and model structure (Appendix S2), graphical plots of structural variables and varied models (Appendix S3), environmental covariates (Appendix S4), graphical plots of model outputs (Appendix S5), results of cross‐validation (Appendix S6), environmental covariates for spatial prediction (Appendix S7), modeling issue of Eq. 2 (Appendix S8), parameter estimates (Appendix S9), and source R codes of the analysis (Appendix S10) are available online. The authors are solely responsible for the content and functionality of these materials. Queries (other than absence of the material) should be directed to the corresponding author. [file COBI-34-194-s001.zip › cobi13370-sup-0001-SuppMat1.docx]

This is a Supplementary file of following manuscript:

Title: A spatially explicit empirical model of structural development processes in natural forests based on climate and topography

There are two other supplementary files: one is the results of the analysis (Excel file), and another is the statistical code used in R.

Appendix S1. National Forest Inventory (NFI) data.

NFI in Japan

The survey of national forest inventory started in 1999, and five years were required to complete a survey of all plots. All plots have since been surveyed three times, and we used all the available data (first to third periods data) which were collected during 1999-2013. Surveys of the NFI plots record species and DBH for trees with >18 cm, >5 cm, and >1 cm DBH in 0.06 ha, 0.03 ha, and 0.01 ha plots, respectively, with smaller plots nested within larger plots (Hirata et al. 2016). In every plot, slope aspect and angle, forest coverage, height of 20 trees (using height pole, vertex, range finder, etc.), dominant tree species, successional stage, and coverage of each stratum (canopy tree, sub-canopy tree, shrub, and herb) are recorded. Rock types are retrieved from the geological map of Japan and soil types are assessed at the site. Forest type (natural forests, plantations, non-forests, etc.) and stand age are derived from the forest registers.

Treatment of NFI data

When planted conifer trees fail to mature, non-planted broad-leaved trees regenerate and the distinction in forest structure and composition between plantations and natural forests becomes blurred (e.g., Hasegawa and Taira 2000). This can be problematic when we treat such ‘unsuccessful’ plantations as natural forests irrespective of the initial applications of silvicultural practices. To reduce this ambiguity, for natural forests, we used only those data recorded as natural forests in forest registers and classified as natural forests during the field survey (*n* = 5,651). In this process, we excluded plots including natural forests if they also included non-forests (e.g., rocks) and plantation forests. Although we used the stand age in forest registers, there were plots whose ages were not updated after recent timber harvesting. We therefore excluded plots (i) with no trees >1 cm DBH, (ii) classified as the early-successional stage (usually <15 years old), and (iii) ≥ 20 years old (*n* = 12). We also excluded incomplete data: (i) without stand age information (*n* = 57), (ii) without tree species identity (*n* = 4), or (iii) ≥ 300 years old (due to the scarcity of quite old forests) (*n* = 3). We note that, in Japan, when forests are harvested, forest registers are updated and therefore information on stand ages are also updated. Forest registers are updated every five years by local governments.

For any of focal structural variables, we excluded outliers, which are due to the possible measurement and recording errors, deviating by >6.5 times of SDs from mean values (*n* = 25), which yielded 5,550 plots. Furthermore, before the analysis, we excluded the data with zero solar radiations or no snow depth information (i.e., NA) due to the erroneous localization (they are usually on the open water: *n* = 44) (resulted in 5,506 plots). We finally used NFI data within the range of 45-180 warmth index to exclude subalpine and subtropical forests. Analyzed data were composed of 4,729 plots for the third period. For the first and second periods data, we only used the plot data that were surveyed in the following (one-more recent) periods as continued surveys and when differences in the stand age between two terms were 0 to 9 years (obtained by recent stand age – older stand age). Since the same plots are designed to be surveyed every five years, by this screening process, we excluded the plot data whose stand age were not accurate. Although this process can also exclude the older data whose plots incurred the harvesting (they take the minus age differences), only small number of the plot data met this condition and therefore was excluded. As an example, for the second period, 184 plots were excluded by this criterion while 299 plots were excluded since the age difference was larger than 9 years. We also excluded the plot data that were not surveyed at the third period as continued surveys since the accurate geographical coordinates measured by geographical positioning system (GPS) have been reported since the third period. Since we examined the effects of terrain covariates derived from digital elevation model (DEM) and geographical information system (GIS), every site is needed to have accurate geographical coordinates. By using this site restriction rule, we presumed that plot data collected at the older two periods were at the coordinates reported at the third period. The final data set was composed of 9,244 plot data (Appendix S1A).

Appendix S1A. Data structure before/after the restriction based on stand age.

|  | Before restriction | Finally used |
| --- | --- | --- |
| Period 3 | 4,729 | 4,729 |
| Period 2 | 3,064 | 2,581 |
| Period 1 | 2,508 | 1,934 |
| Total | 10,301 | 9,244 |


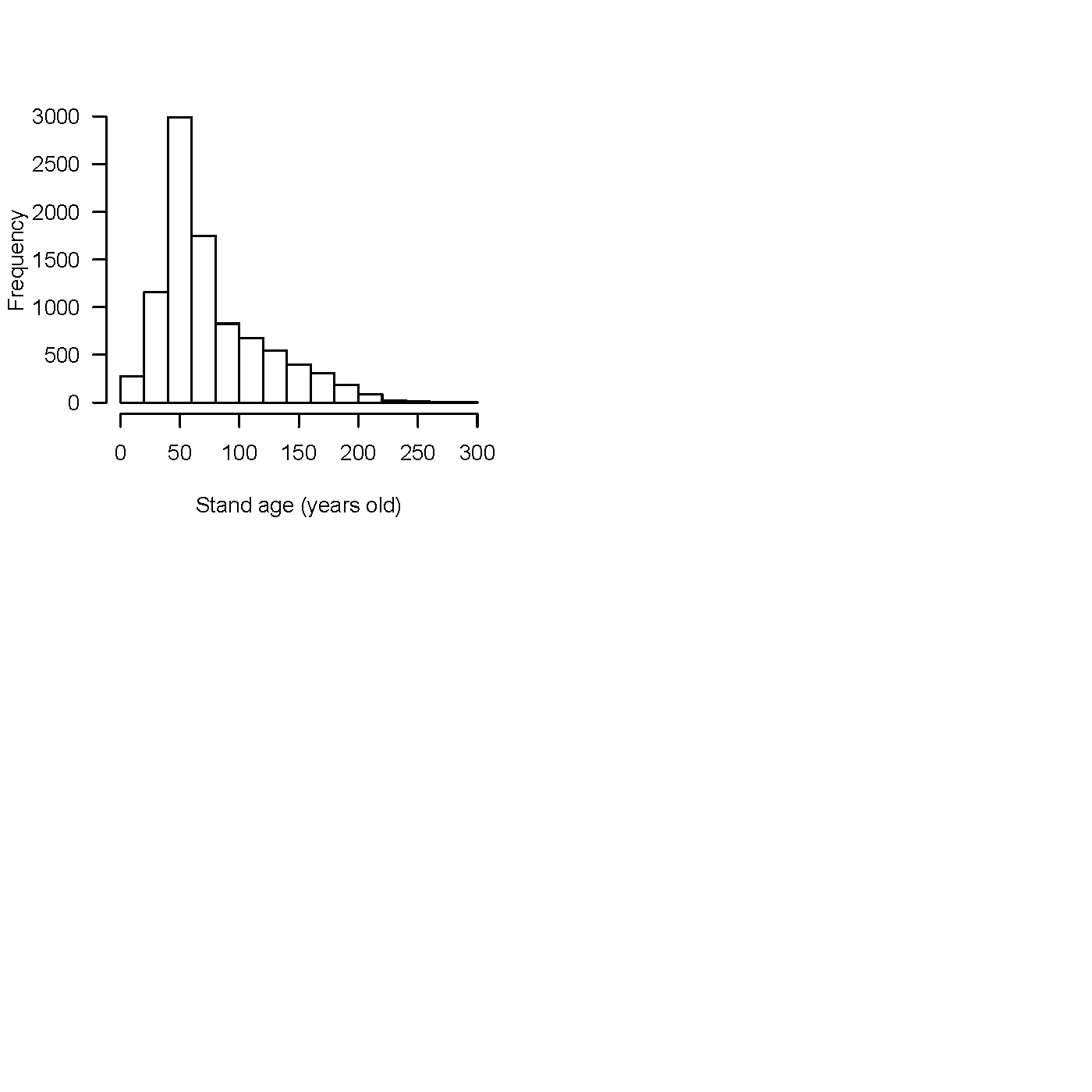


Appendix S1B. Histogram of stand age for analyzed plot data (*n* = 9,244).

Appendix S2. Treatment of mean DBH, tree density, and plot data of old-growth forests.

The old-growth index was a composite of individual structural variables. Structural variables were scaled (with range of 0-1) using the values of young stands from NFI data and those of qualified old-growth forests in Japan (see Methods section in the main text for details). Here, we describe the relevant information about this procedure.

Tree density

Although mean DBH, large tree density and SD of DBH increased monotonically with stand age, tree density showed a more complex, unimodal response to stand age (Appendix S3A). Values for tree density (based on live trees with >5 cm DBH) were limited immediately after timber harvesting, then increased, peaked at ~30 years old, and then decreased with the age. Therefore, there was limited difference in median values of tree density between young forests (≤10 years old) and old-growth forests (Appendix S3A). Although we could increase these differences using the plots supporting stands that were ≤20 or ≤30 years old to obtain values for tree density for young forests (Appendix S3A: 1,753 [≤20 years old]; 2,000 [≤30 years old]), the modeled environmental dependency of the development rates was similar irrespective of the thresholds (Appendix S3C).

Mean DBH

Some values of mean DBH in stands ≤20 years old had a relatively high value (>20 cm); we therefore used 30 years old to define young forests for mean DBH. This change avoided the inflation of the intercept of the development model (Eq. 2).

Large tree density

We used the threshold of 40 cm to define large trees since values larger than 40 cm diminished the among-plot differences (Appendix S3P).

Coarse woody debris as a structural variable

We also did not use the coarse woody debris (CWD) since the registered permanent plot data in Japan only include live trees (Ishihara et al. 2010).

Correlation among the three structural variables

Above described four structural variables had |0.31|–|0.73| correlations each other while the correlations slightly increased after the logit-transformation (Appendix S3M-N).

A hierarchical model to accommodate stand age differences

In their analysis of structural development rates, Larson et al. (2008) modeled structural development processes at 12 permanent plots individually. They then calculated the development rates for individual plots and identified relationships between the structural development rate and site productivity a posteriori. However, this approach requires plot-specific longitudinal data (≥48 years in their study), and it is difficult to quantify the spatial effects of environmental factors on structural development rates. We instead used NFI data from a range of environments. However, although we used all available NFI data collected from 1999-2013, these data are snap-shot data, and survey plots differ in stand age (Fig. 1a). This means that, even if the different plots were to have the same structural development rates, they would have different levels of structural development due to differences in stand age and vice versa. Therefore, an analysis that fails to incorporate stand age can produce misleading results. We dealt with these issues by using hierarchical Bayesian inference (Gelman and Hill 2007). Specifically, we treated unobserved development processes in which the observed states (measured stand structure) appeared as latent (hidden) processes (sensu Royle and Dorazio 2008). The combination of Bayesian inference and NFI data facilitated the modeling of development rates as a function of environmental covariates derived from GIS.

Low-rank thin-plate splines

To model possible more complex functional responses of the development rates to environmental covariates, we also constructed the low-rank thin-plate splines (one of the generalized linear models) by replacing Eq. 3 and fitted the developed models to the data. We modeled the smoother as a straight line (linear model) and a set of cubic terms with nine knots based on sample quantiles, following Crainiceanu et al. (2005) and Zuur et al. (2012). We note that even when we included the quadratic term into the smoother (rather than linear model), results were similar. Our model of spline function can be represented as follows:

$coeff=\beta_{1}\boldsymbol{+\beta}\times\boldsymbol{X}+\boldsymbol{b}\times\boldsymbol{Z}$, Eq. (S1)

where *β*_1_ is an intercept, ***X*** and ***β*** represent six environmental covariates and associated regression parameters (only composed of linear terms), respectively, and ***Z*** and ***b*** represent cubic terms of six covariates and associated coefficients, respectively. ***Z*** contains nine cubic terms of six covariates (totally, 54 terms = 9 × 6). As an example of simple notation, for a certain covariate of the *j*th site, cubic terms and their coefficients are *b*_1_ × |*x_j_* – *K*_1_|^3^ + *b*_2_ × |*x_j_* – *K*_2_|^3^ + … + *b*_8_ × |*x_j_* – *K*_8_|^3^ + *b*_9_ × |*x_j_* – *K*_9_|^3^ where nine terms of the *K*s are the knots based on nine sample quantiles (10%, 20%, …, 80%, 90%).

Results showed that modeled environmental dependency of the development rates by splines was similar with that modeled by simple quadratic models (Appendix S3G-H). However, their confidential intervals were wide, and most environmental effects were not significant. We therefore simplified the spline models, that is, we constructed five knots splines. Although the modeled functional forms were similar, most effects were again not significant (Appendix S3I-J). Larger numbers of regression parameters of splines may lead to difficulty to find the significant environmental dependency of the development rates (in terms of random slopes) given the information of NFI tree plot data. We therefore used simpler quadratic models in this study.

Appendix S3. Structural variables from NFI data and permanent plot data.


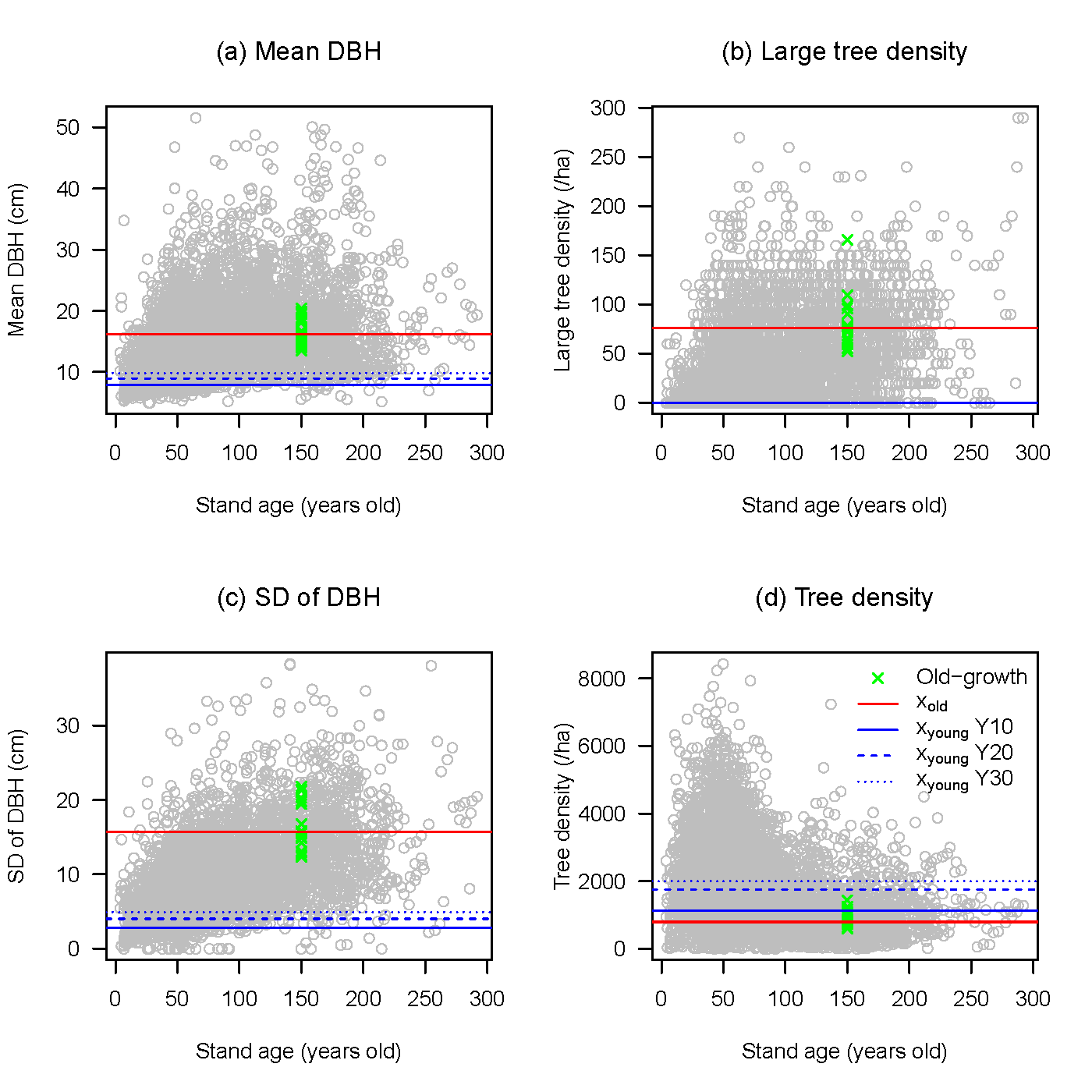


Appendix S3A. Four structural variables in relation to stand age in natural forests.

Eighteen values from qualified old-growth are shown at 150 years old. Median values of old-growth and young forests are indicated by the horizontal lines. For young forests, three different values with different threshold years (≤10, 20, and 30 years old) are shown by different line types. See Appendix S3T for the specific values used in the analysis to characterize young and old-growth forests.

Appendix S3B. Four structural variables in relation to forest types of 29 qualified old-growth forests in Japan.

Abbreviation: BC (mixed forest composed of broad-leaved and conifer tree species); DB (deciduous broad-leaved forest); EB (evergreen broad-leaved forest); EC (evergreen coniferous forest). Numbers below the forest types (only in *a*) indicate the number of plots. Note that BC and EC forests comprise forests across various climatic zones (e.g., subalpine zones). Although there were 15 old-growth forests registered as ‘DB’, one plot (OZ-DB1) was excluded since the plot was dominated by a coniferous species *Thujopsis dolabrata*.

Appendix S3C. Modeled environmental dependency of development rates of live tree density.

Results with different age thresholds to define young forests are shown. Real and dotted lines indicate posterior means and 95% confidential intervals, respectively.

Appendix S3D. Modeled dependency of forest development rates on six environmental covariates. We produced these plots by excluding live tree density to examine the effects of excluding live tree density on the results of old-growth index. See *Model structure* of Statistical analysis in the main text.

Appendix S3E. Environmental dependency of development rates modeled by low-rank thin-plate splines based on nine knots of sample quantities.

Appendix S3F. Environmental dependency of development rates modeled by low-rank thin-plate splines based on five knots of sample quantities.

Appendix S3G. Environmental dependency of development rates modeled by quadratic model.

Real and dotted lines indicate posterior means and 95% confidential intervals, respectively.


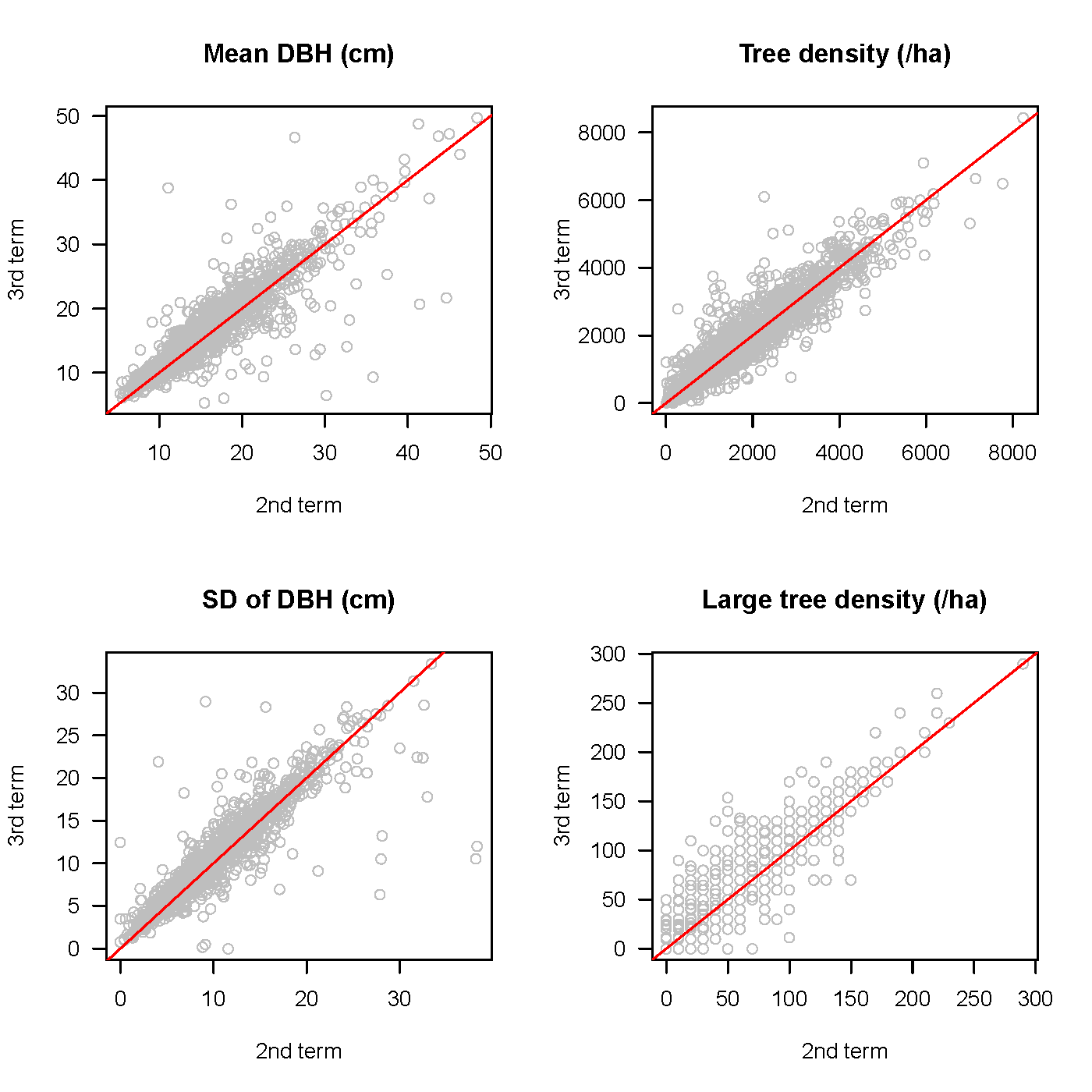


Appendix S3H. Comparison of four structural variables between third and second periods (terms) NFI data.

Red lines indicate 1:1 lines (*n* = 2,581).


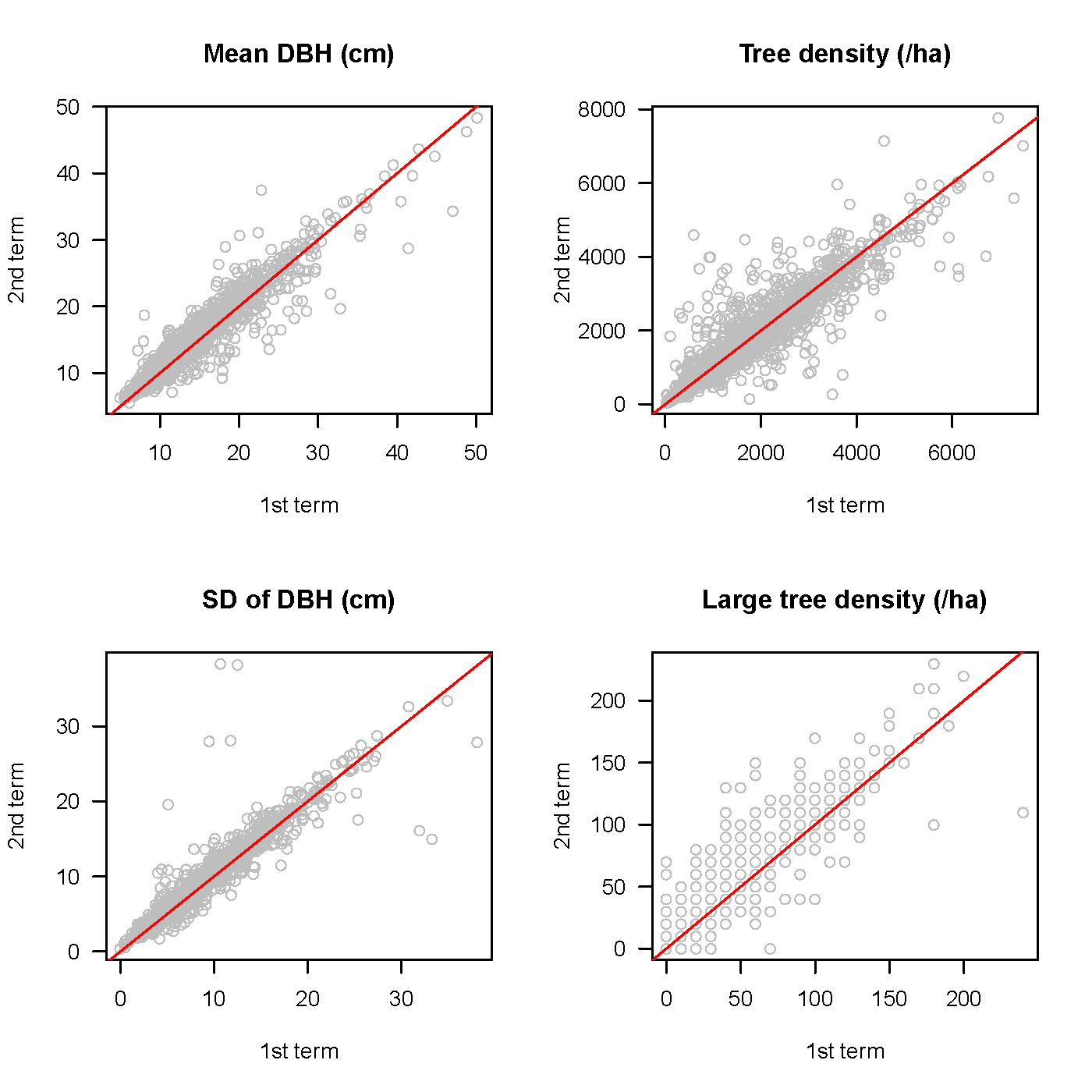


Appendix S3I. Comparison of four structural variables between second and first terms NFI data.

Red lines indicate 1:1 lines (*n* = 1,934).


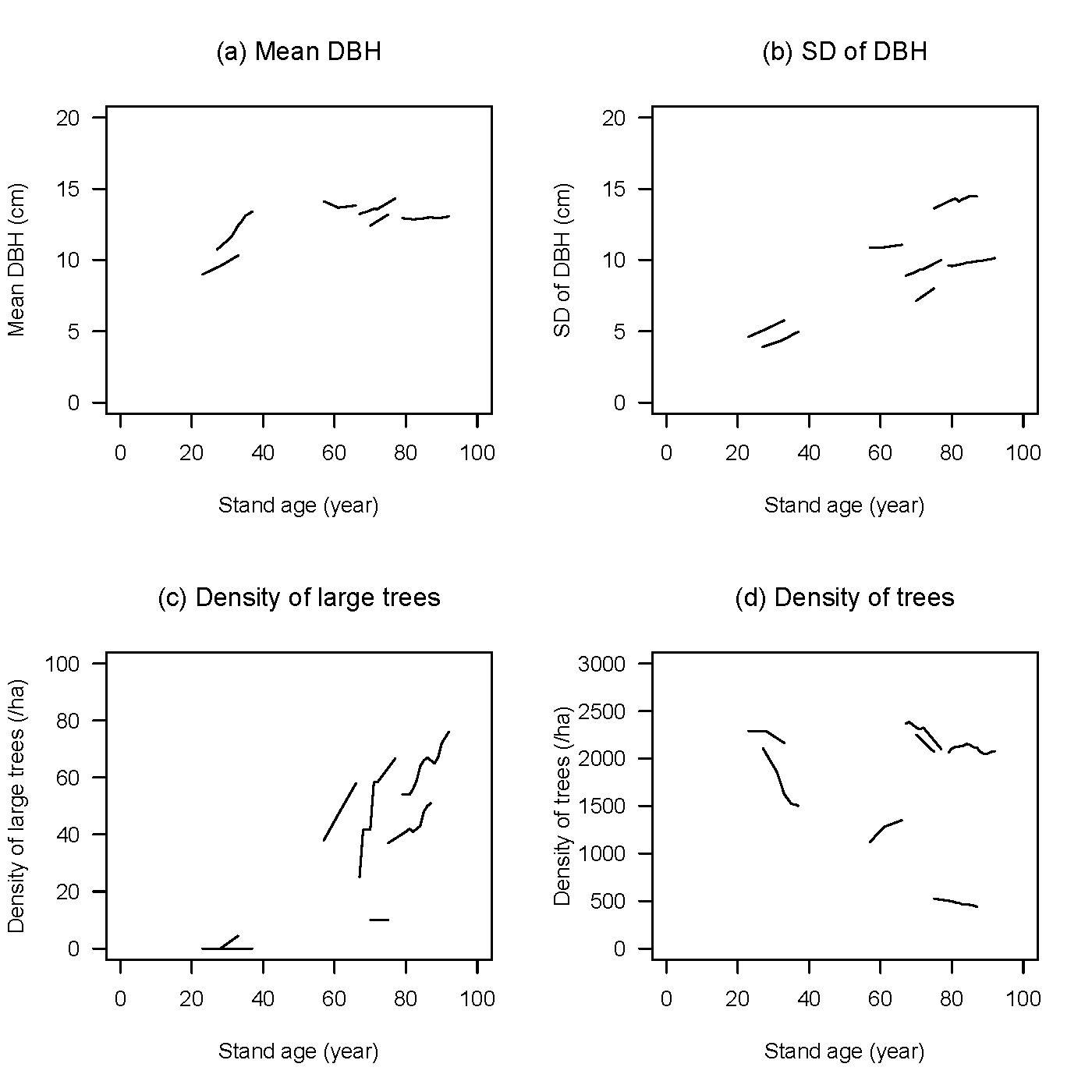


Appendix S3J. Temporal changes in four structural variables in seven permanent plots of Japanese broad-leaved forests.

Appendix S3K. Large tree densities with different thresholds.

| Threshold | Mean | SD |
| --- | --- | --- |
| 20 cm | 237 | 145 |
| 30 cm | 82 | 71 |
| 40 cm | 29 | 37 |
| 50 cm | 12 | 20 |
| 60 cm | 5 | 12 |

Density: trees per ha.

We calculated large tree density by different thresholds, and finally used trees with >40 cm DBH in the analysis. Large trees were rare across the plots and the use of >40 cm DBH threshold diminished the variation among the plots.


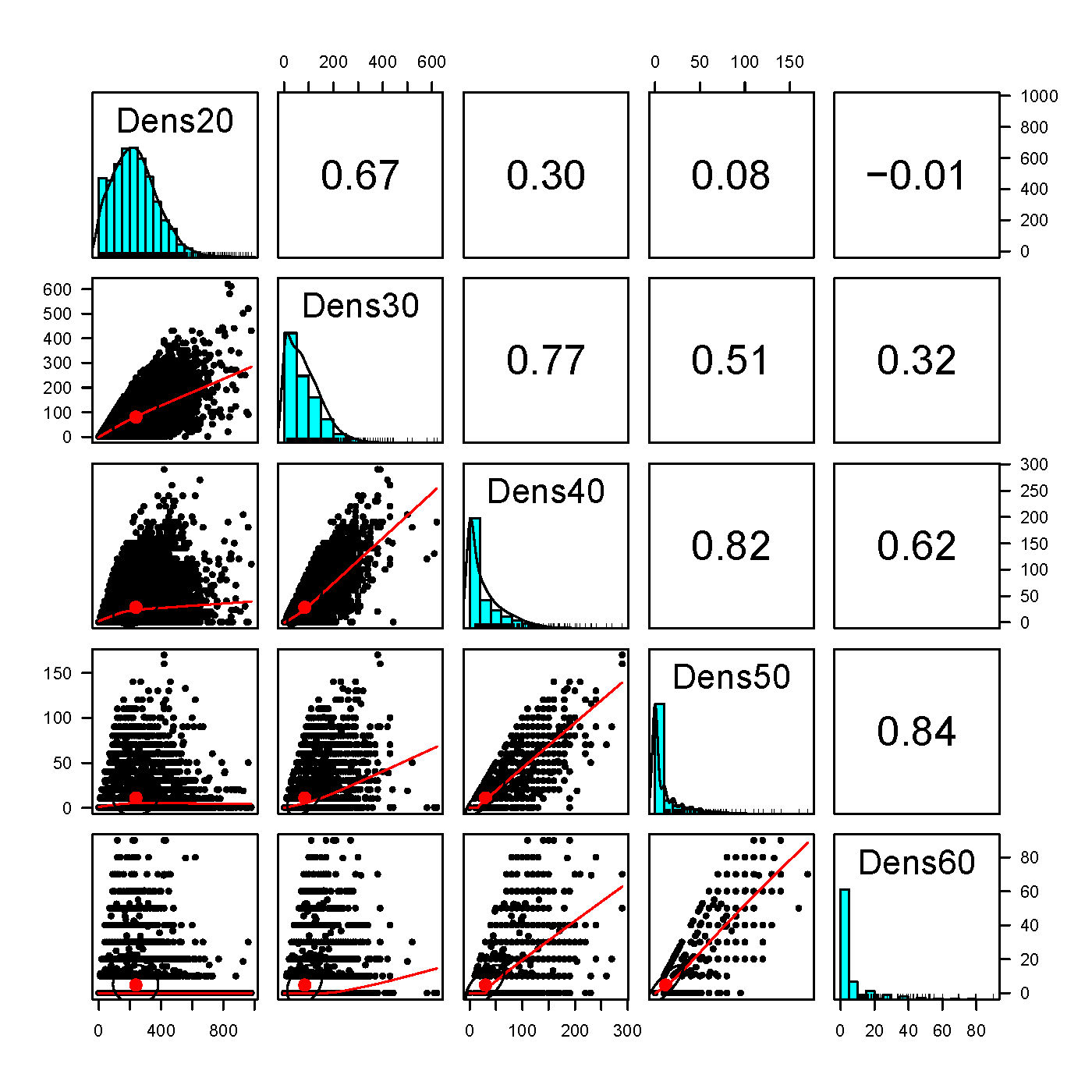


Appendix S3L. Correlation plots of large tree densities with different thresholds.

Plots are created ‘pairs.panels’ function of ‘psych’ R library with default setting. Panels below the diagonal show scatter plots of the pairs of large tree densities with different thresholds (with loess regression lines). Pearson correlation values of the pairs are shown above the diagonal while histograms of individual densities are shown at the diagonal.


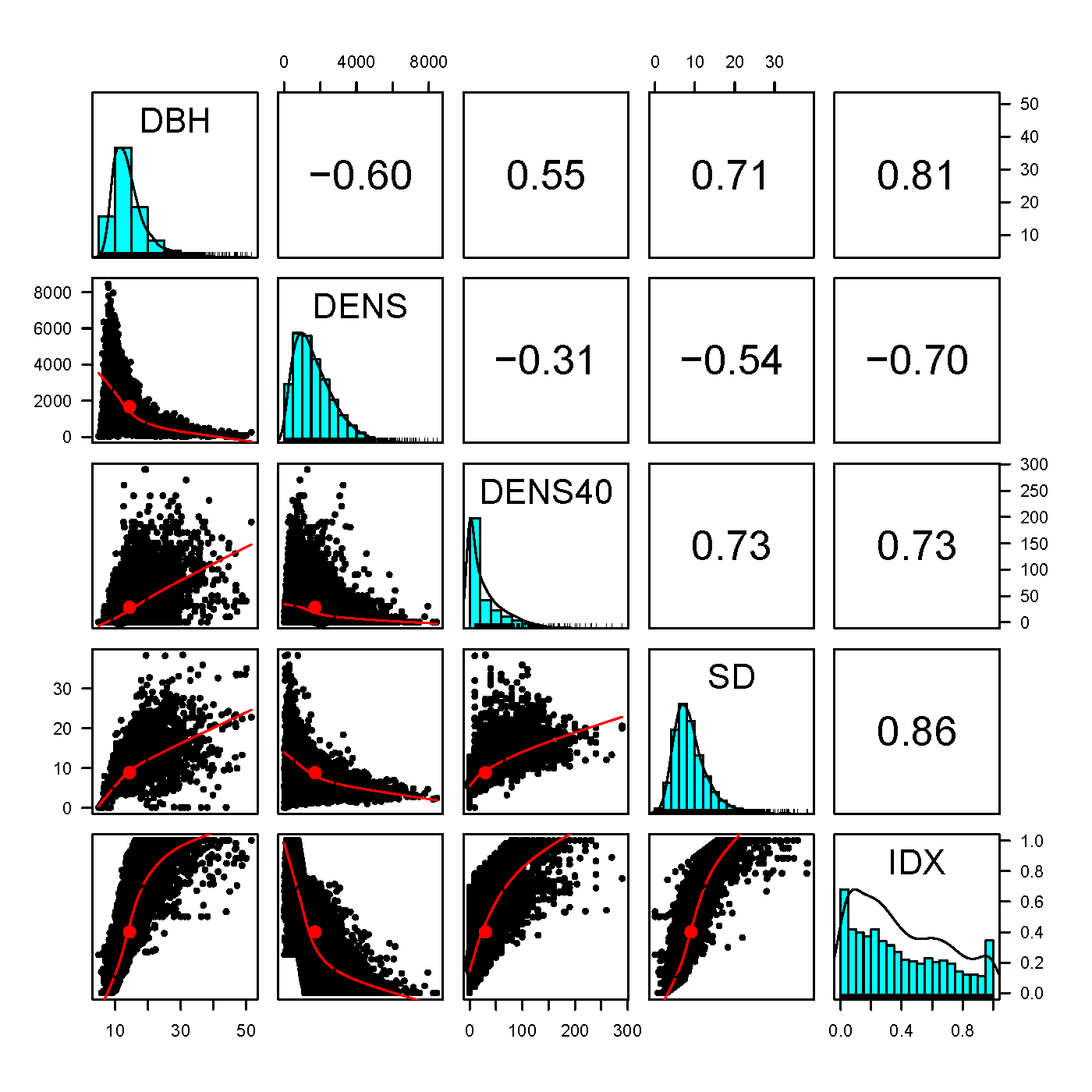


Appendix S3M. Correlation plots of structural variables and old-growth index.

Abbreviations: DBH (mean diameter at breast height [cm]); DENS (live tree density [/ha]); DENS40 (density of large tree [>40 cm DBH] / ha); SD (standard deviation of DBH [cm]); IDX (old-growth index).


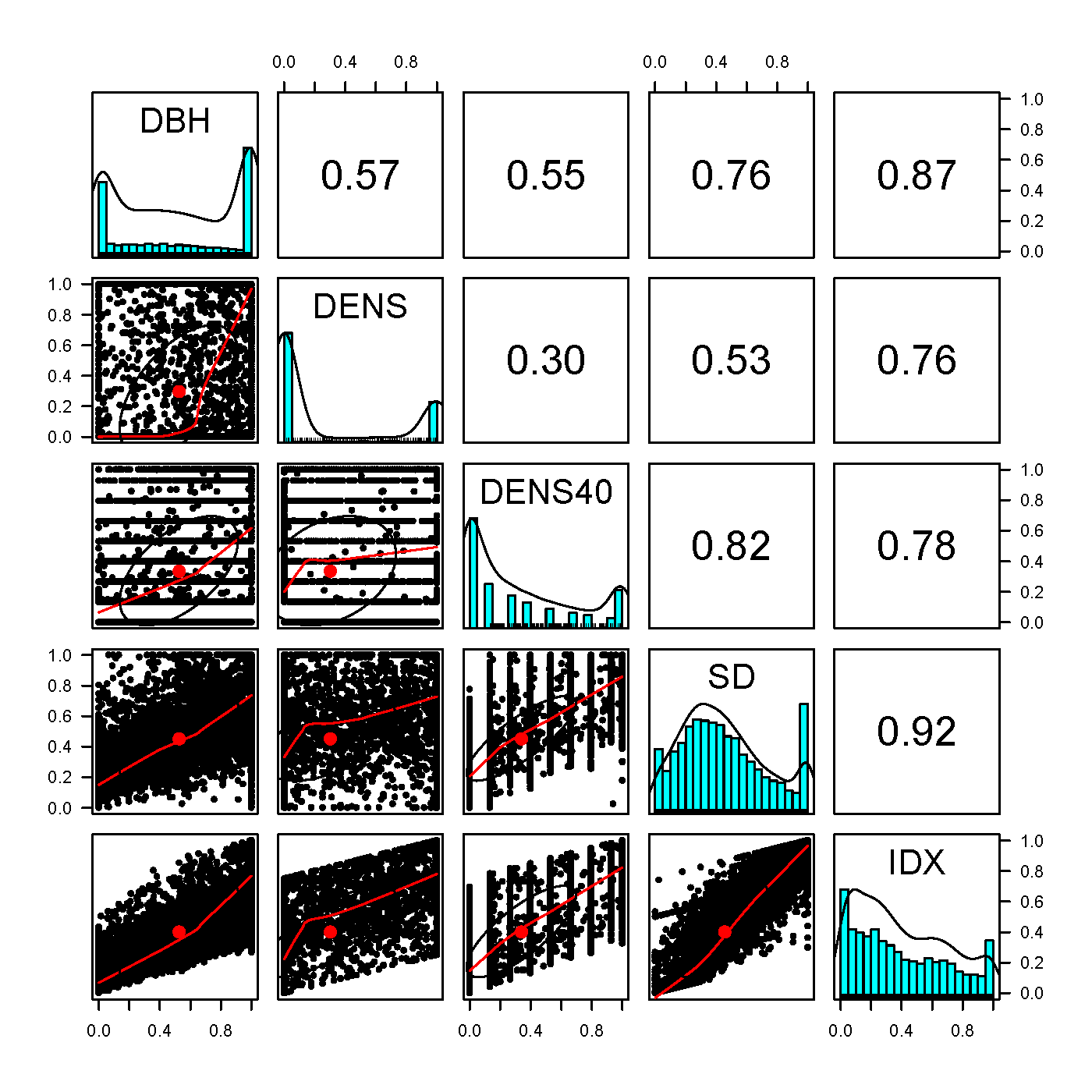


Appendix S3N. Correlation plots of structural variables and old-growth index (after logit-transformation).

See Appendix S3R for the abbreviations.

Appendix S3O. Conditional values used for the construction of the index.

| Variable | Age_young_^a^ | *x*_young_^b^ | *x*_old_^c^ | ε_lower_^d^ | ε_upper_^e^ |
| --- | --- | --- | --- | --- | --- |
| Mean DBH (cm) | 30 | 9.8 | 16.2 | 0.001 | 0.0003 |
| Live tree density (/ha) | 10 | 1135 | 797 | 0.004 | 0.003 |
| Large tree density (/ha) | 10 | 0 | 76 | 0.13 | 0.001 |
| SD of DBH (cm) | 10 | 3 | 16 | 0.0007 | 0.0005 |
| *I*_og_ | NA | NA | NA | 0.0001 | 0.0001 |

^a^ Stand age threshold to define younger forests to represent their states (*x*_young_). See Appendix S2 for the treatment of mean DBH.

^b^ Median value of structural variable for young forests.

^c^ Median value of structural variable for 18 old-growth forests across Japan.

^d^ Minimum non-zero value added to the lowest value (0) for the logit-transformation.

^e^ Minimum non-zero value subtracted from the largest value (1).

Appendix S4. Treatment of the climate and topographic covariates.

The data set ‘Climate Mesh Data 2000’

The data set of ‘Climate Mesh Data 2000’ contained average values of daily high, low and average temperature, precipitation, maximum depth of snow cover, and quantity of solar radiation for every 1 km mesh (or grid) in Japan from 1971 to 2000. Values for snow depth and warmth index were then calculated for each survey plot of NFI. Although snow cover represents the winter environment rather than that of growing period, it can affect tree survival and regeneration processes by such as causing trunk bending and mechanical injury as well as by reducing seed predation by rodents (Homma 1997, Homma et al. 1999).

Treatment of digital elevation model

We resampled the 20-m digital elevation model (DEM) from a ‘10-m’ DEM published by Geospatial Information Authority of Japan, which was interpolated from 10 m interval contours of the 1:25 000 topographic maps. Since the grid-point interval of the original ‘10-m’ DEM was measured in degrees in the longitudinal and latitudinal directions, cubic convolution was applied for resampling, using Universal Transverse Mercator coordinate system. We used a spatial resolution of 20 m for reasonable computation time in the whole Japan without losing significant local topographic features. We obtained topographical values for the plots using the bilinear interpolation of four pixels nearby each plot.

We computed slope angle as the maximum rate of elevational change between each pixel and its neighbors. For describing vertical topographic exposure, we calculated positive topographic openness (Yokoyama et al. 2002) within 100 m radius of each pixel. For describing hydrological conditions, we computed specific catchment area using the D-infinity flow direction algorithm (Tarboton 1997). Potential solar radiation at each plot was computed by measuring solar incident angles at each site in every fifteen minutes in every two weeks from January to December in 2017 with a sky size (i.e., viewshed distance from each plot) of 2 km.

We initially considered the use of topographic wetness index (TWI) as an indicator of soil moisture and nutrients rather than upslope catchment area:

TWI = ln (*A* / tan *β*)

where *A* is upslope catchment area and *β* is slope angle.

However, since our mapping area (shown in Fig. 4) includes flat areas and it was difficult to accurately obtain slope angle and TWI in such areas based on DEM, we did not use TWI but the catchment area. Nevertheless, we note that TWI and log-transformed catchment area were highly correlated each other (*r* = 0.87).

We also note that in our preliminary analyses, we grouped soils and geology of the plots into eight soil and four rock types, respectively, but this explained only a small amount of variation in structural variables. We therefore did not further consider these covariates.

Model selection

We did not undertake model selection. This was first because many covariates had significant effects given the large sample size and second because we compared the fitted functional forms of the structural variables. Although some of the parameter estimates were not significant in the full model (Appendix S9), their inclusion in the model did not produce adverse effects. For example, the model of the old-growth index included three (out of 14) non-significant parameters: a quadratic term for the slope angle and simple and quadratic terms for terrain openness. Graphical presentation of the modeled functional form showed that slope effects on the index was linear and the effects of terrain openness were quite weak (Fig. 2, Appendix S3G). We also constructed a more parsimonious model for the old-growth index without non-significant terms in the full model, and it yielded quite similar parameter estimates to the full model (Appendix S9G). In this study, we therefore did not undertake model selection. Nevertheless, we acknowledge that more parsimonious models with fewer covariates would be useful in many practical situations.

Appendix S4A. Environmental covariates used in the analysis.

| Covariate | Description | Mean | SD | Min | Max |  | Data source |
| --- | --- | --- | --- | --- | --- | --- | --- |
| *Stand* |  |  |  |  |  |  |  |
| Stand age | Age of surveyed plots | 77 | 44 | 4 | 292 |  | NFI |
|  |  |  |  |  |  |  |  |
| *Climate* |  |  |  |  |  |  |  |
| Snow depth | Maximum snow depth (cm) | 78 | 79 | 0 | 503 |  | Climate Mesh Data 2000 |
| Warmth index | Cumulative temperature of months above 5°C | 78 | 25 | 45 | 177 |  | Climate Mesh Data 2000 |
|  |  |  |  |  |  |  |  |
| *Topography* |  |  |  |  |  |  |  |
| Slope angle | Measured slope at surveyed plots (°) | 26 | 12 | 0 | 62 |  | NFI |
| Positive topographic openness | Openness of the terrain to the sky (°) | 83 | 7 | 56 | 105 |  | DEM |
| Catchment area | Upslope area where precipitation collects into plots (ha) | 1 | 2 | 0 | 60 |  | DEM |
| Potential solar radiation | Intensity of solar radiation throughout a year | 1,198,782 | 205,387 | 454,106 | 1,755,744 |  | DEM |

Source data comprises NFI data and permanent plot data.


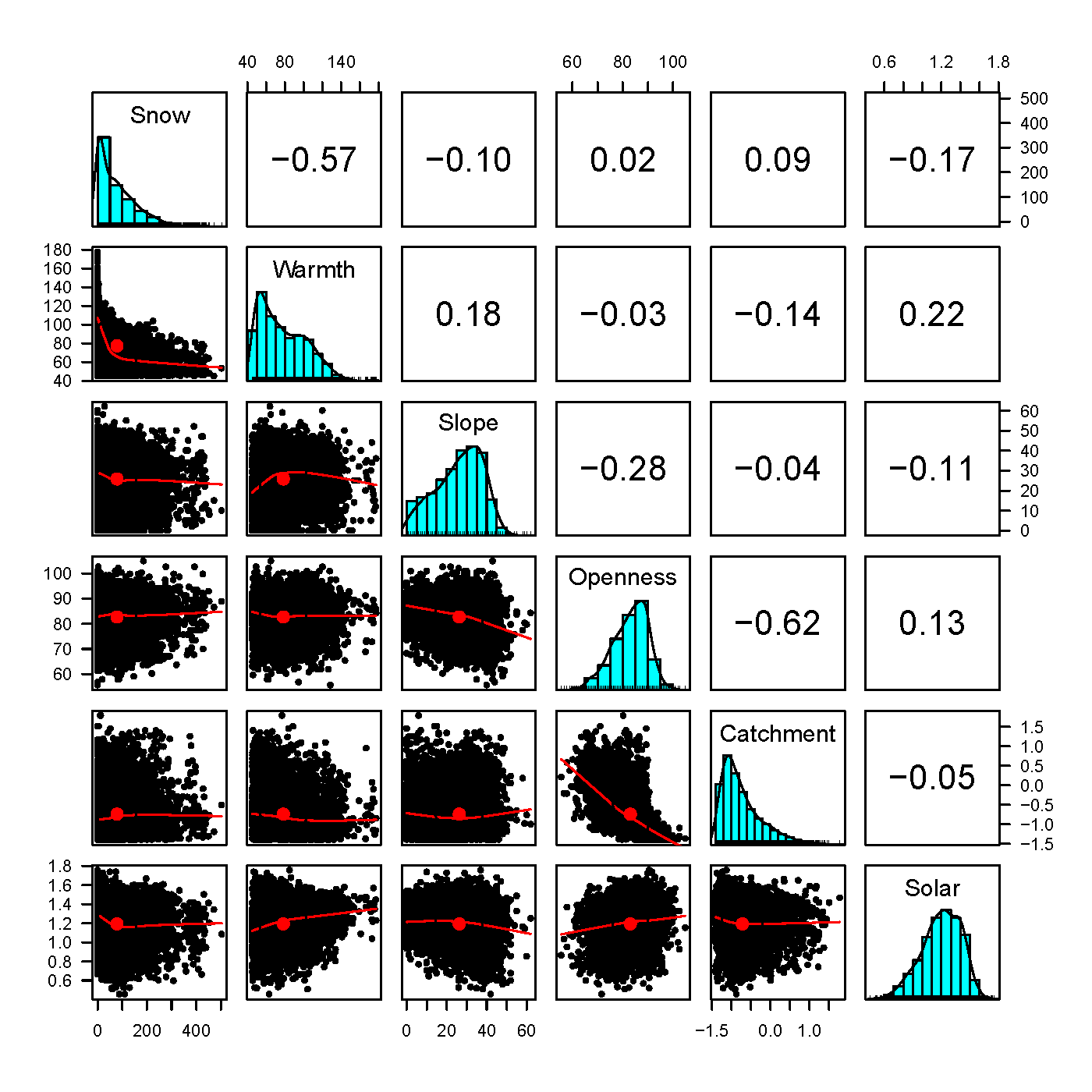


Appendix S4B. Correlation plots of six environmental covariates.

Parameters: snow depth (cm); warmth index; slope angle (°); terrain openness (°); catchment area (log_10_[ha]); solar radiation.


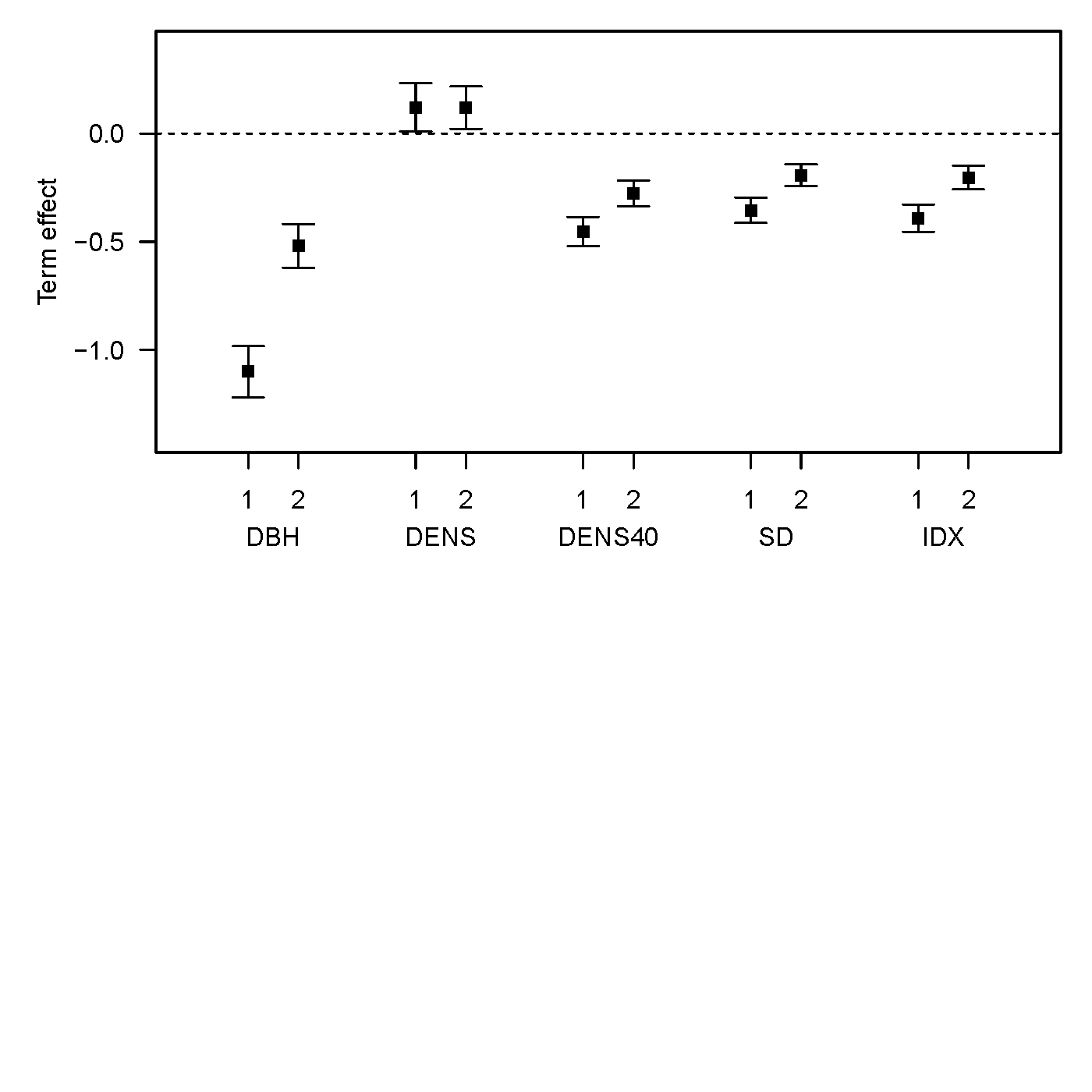


Appendix S4C. Effects of measurement period on structural variables and old-growth index.

NFI plots were measured three times and we modeled the effects of first and second measurement periods (terms) relative to the third period in Eq. 2. Positive and negative effects indicate that older periods were inclined to yield larger and smaller indices than the latest three period, respectively. Abbreviations: DBH (mean diameter at breast height [cm]); DENS (live tree density); DENS40 (density of large tree [>40 cm DBH]); SD (standard deviation of DBH); IDX (old-growth index). Since tree density (DENS) reduces with stand age, the positive effects means that tree densities measured by older periods were smaller than those of the third period after accounting for the differences in stand age given the model structure (see also Appendix S3M-N).

Appendix S5. Modelled old-growth index as the function of climatic and topographic covariates.

Appendix S5A. Inferred logistic curves for mean DBH of natural forests against stand age.

Curves with different covariate values (within the 95% percentiles) are depicted. For example, in Appendix S5Ab, four fitted lines with four different values of warmth index (0.025 and 0.975 quantiles as well as their intermediate values) are shown. Other covariates were held constant at mean values.

Appendix S5B. Inferred logistic curves for live tree density of natural forests against stand age.

Appendix S5C. Inferred logistic curves for large tree density of natural forests against stand age.

Appendix S5D. Inferred logistic curves for SD of DBH of natural forests against stand age.

Appendix S5E. Graphical examination of prediction errors for mean DBH.

(Top-left) Observed values and prediction values are plotted. (Top-right) Observed (grey color) and predicted (blue) values are plotted in relation to stand age. (Bottom-right) Prediction errors (observed values – predicted values) are plotted in relation to stand age. Histograms of observed values, prediction values, and prediction errors are also produced. Predictions were made with measurement period effects but without random site effects.

Appendix S5F. Graphical examination of prediction errors for live tree density.

See Appendix S5E for details.

Appendix S5G. Graphical examination of prediction errors for large tree density.

See Appendix S5E for details.

Appendix S5H. Graphical examination of prediction errors for SD of DBH.

See Appendix S5E for details.

Appendix S5I. Graphical examination of prediction errors for old-growth index.

See Appendix S5E for details.

Appendix S6. Coefficient of determination for the analysis of four structural variables and old-growth index.

|  |  | Cross-validation** | | | | |
| --- | --- | --- | --- | --- | --- | --- |
| Variable | Overall* | Mean | SD | 2.5% | Median | 97.5% |
| DBH | 0.23 | 0.23 | 0.02 | 0.19 | 0.23 | 0.27 |
| Live tree density | 0.27 | 0.27 | 0.03 | 0.22 | 0.27 | 0.32 |
| Density of large tree | 0.23 | 0.23 | 0.02 | 0.18 | 0.23 | 0.27 |
| SD of DBH | 0.31 | 0.31 | 0.03 | 0.26 | 0.30 | 0.35 |
| Old-growth index | 0.29 | 0.29 | 0.02 | 0.25 | 0.29 | 0.33 |

**R*^2^ obtained when all data were used for training and testing. **We randomly chose 90% of the data for model training and tested the model using 10% of the remaining data. We repeated this procedure 100 times and obtained the respective statistics of *R*^2^.

Appendix S7. Relevant environmental covariates used for the spatial prediction.


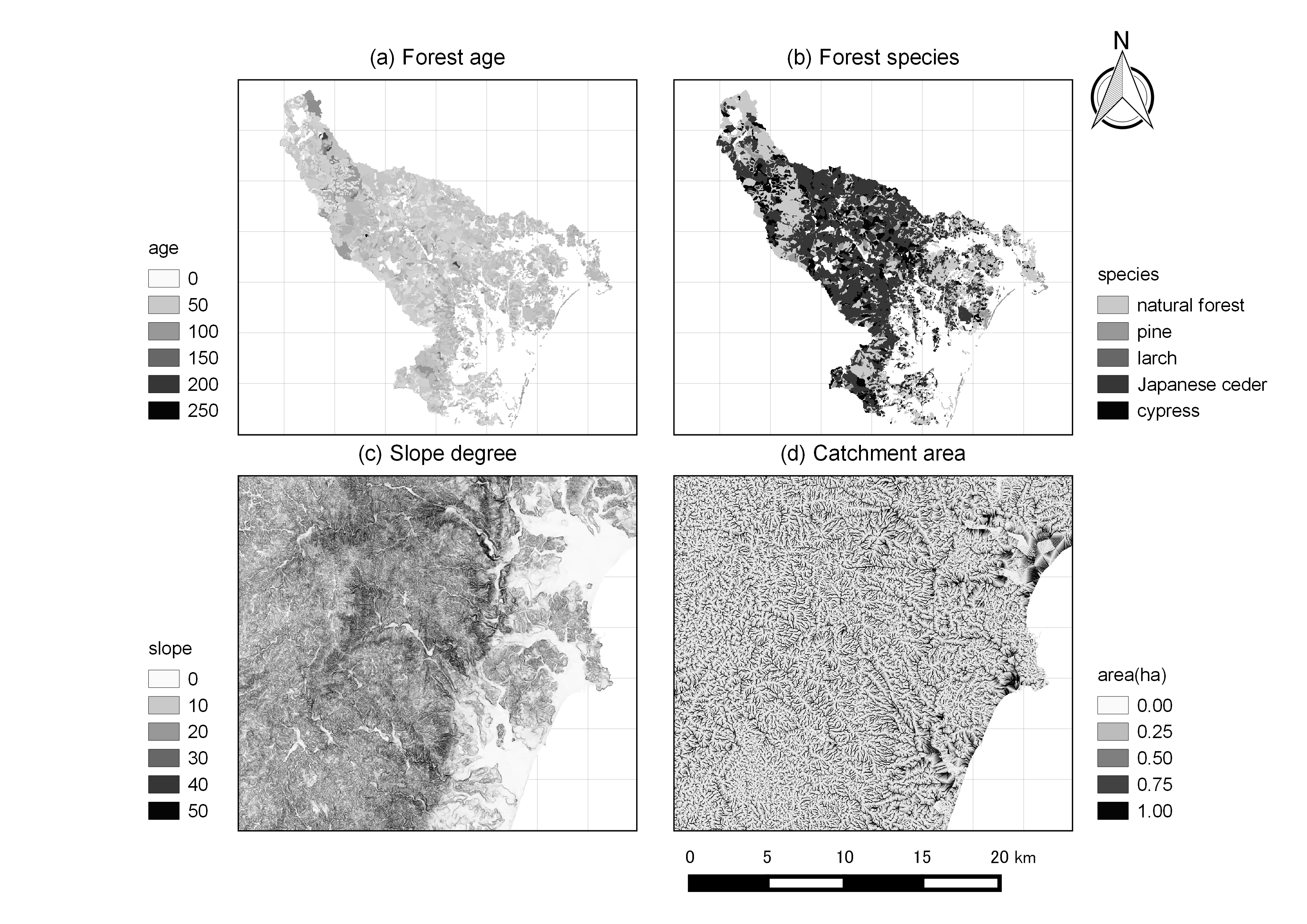


Appendix S7A. Relevant environmental covariates used for the spatial prediction.

Forest register data were only partially available.


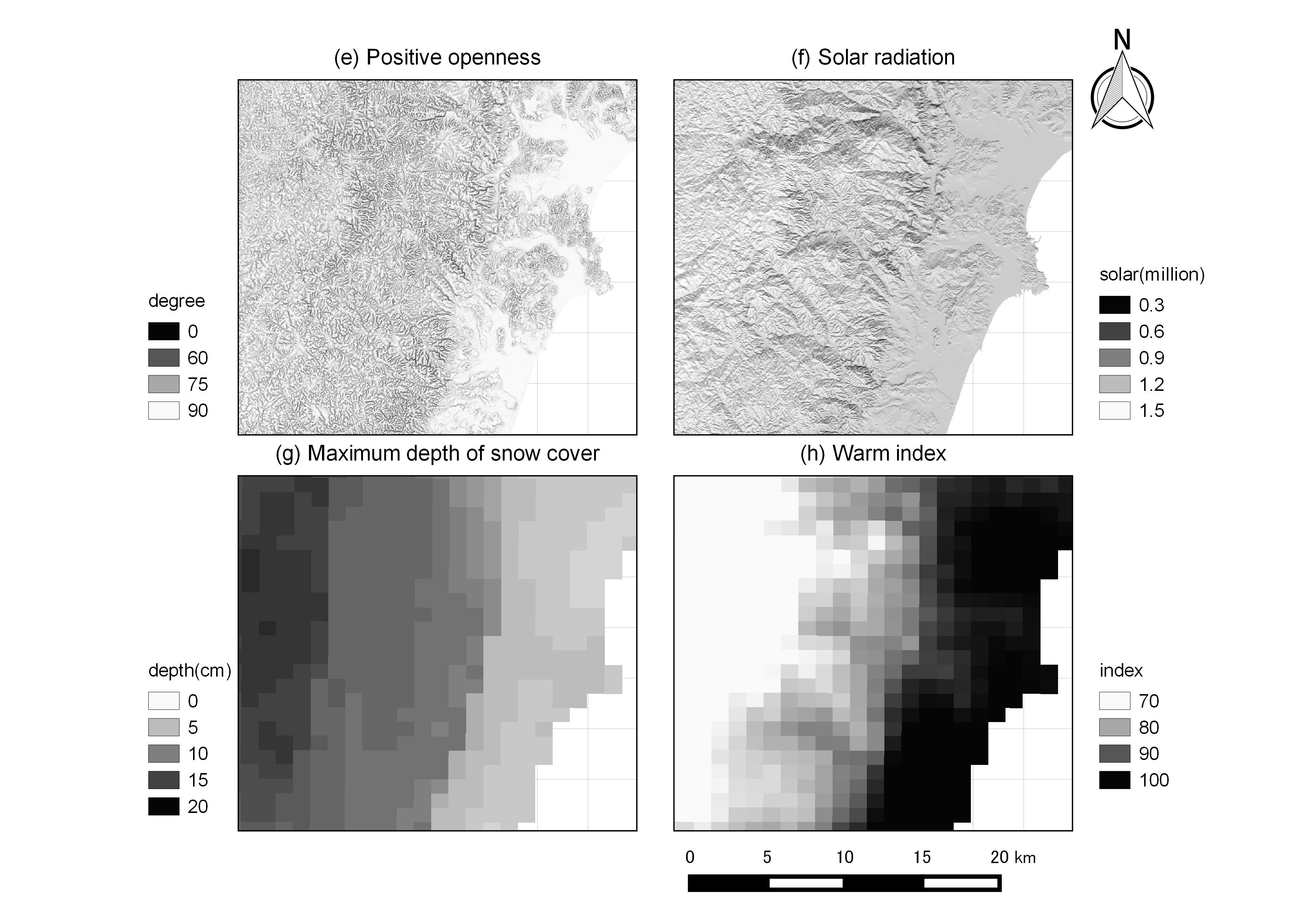


Appendix S7B. Relevant environmental covariates used for the spatial prediction.


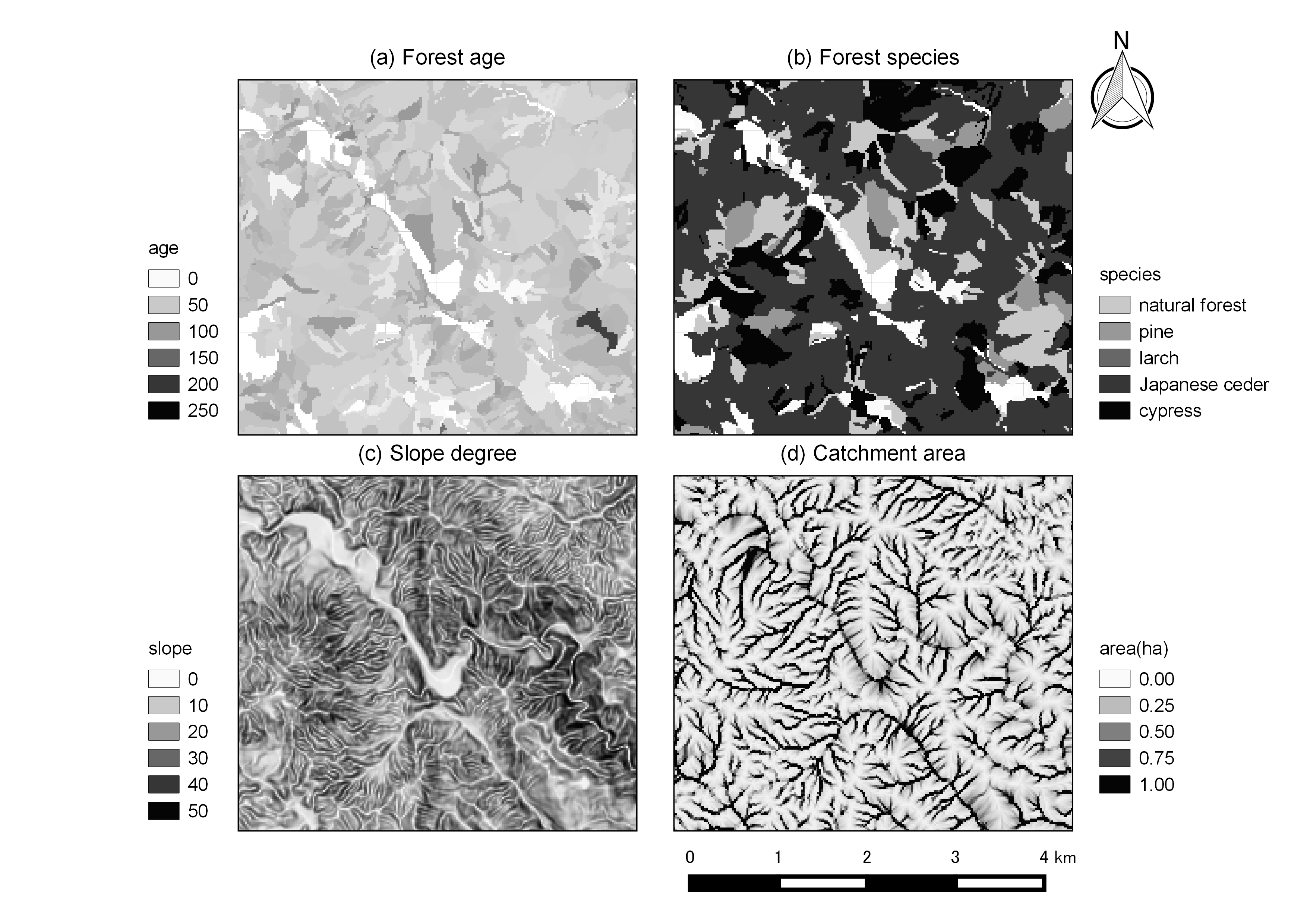


Appendix S7C. Relevant environmental covariates in the central part of the study area.


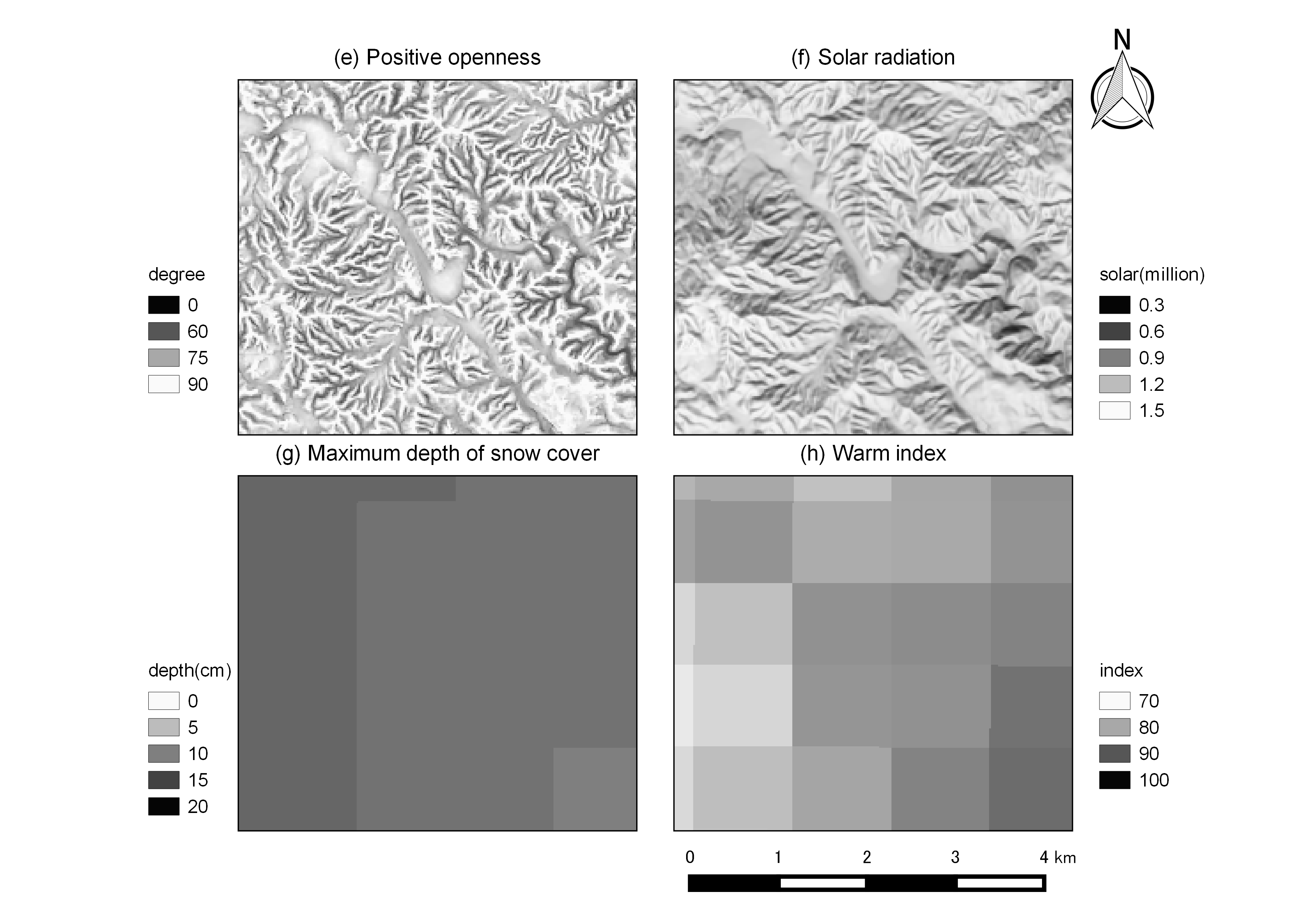


Appendix S7D. Relevant environmental covariates in the central part of the study area.

Appendix S8. Addition of covariates into intercepts.

Larson et al. (2008) assumed that stand development started from clear-cuts and focused on the variability of the development rates rather than the variability of the initial stage. We can consider the variability of the initial stage in relation to environmental covariates by adding the covariates into the intercept-part (*β*_0_) of Eq. 2. In this study, since our primary objective was to develop the modeling framework for the old-growth index proposed by Larson et al. (2008), we made intercept of Eq. 2 constant. The use of constant intercept presumes that structural variables of harvested forests (at zero years old) possessed approximately similar values. We made this assumption because our analyzed plot data were dominated by forests regenerated 40-70 years ago (which is the typical stand age distribution in Japanese natural forests) (Appendix S1B, Yamaura et al. 2012). Natural stands of these ages have been heavily harvested in Japan with many conifer plantations then being established after those harvesting operations (Forestry Agency 2014, Masaki et al. 2017). We then assumed that natural forests would have regenerated from the areas similarly harvested with post-planting, and that natural forests shared approximately similar initial conditions (created by clear-cuts).

Nevertheless, we also fitted an alternative model that incorporated environmental covariates into the intercept of Eq. 2. The results yielded similar values of *R*^2^ and DIC to our previous results. However, some of the parameter estimates in the slopes (Eq. 3) changed; for example, the effect of simple term for catchment area weakened when both the intercept and the slope were hierarchically structured (Appendix S9H). We therefore suggest that future model improvement, including the consideration of initial stand conditions, could be useful.

Reference

Crainiceanu, C. M., D. Ruppert, and M. P. Wand. 2005. Bayesian analysis for penalized spline regression using WinBUGS. Journal of Statistical Software **14**:24.

Forestry Agency. 2014. White paper on forestry and forests, 2013. Japan Forestry Association, Tokyo (in Japanese).

Gelman, A., and J. Hill. 2007. Data analysis using regression and multilevel/hierarchical models. Cambridge University Press, Cambridge.

Hasegawa, M., and H. Taira. 2000. The characteristics of species composition of the hardwoods established on *Cryptomeria japonica* plantation in heavy snow regions. Journal of Japanese Forestry Society **82**:28-33 (in Japanese).

Hirata, Y., K. Hosoda, T. Nishizono, F. Kitahara, I. Nagame, and M. Nakamura. 2016. Japan. Pages 507-520 *in* C. Vidal, I. A. Alberdi, L. Hernández Mateo, and J. J. Redmond, editors. National Forest Inventories: Assessment of Wood Availability and Use. Springer International Publishing, Cham.

Homma, K. 1997. Effects of snow pressure on growth form and life history of tree species in Japanese beech forest. Journal of Vegetation Science **8**:781-788.

Homma, K., N. Akashi, T. Abe, M. Hasegawa, K. Harada, Y. Hirabuki, K. Irie, M. Kaji, H. Miguchi, N. Mizoguchi, H. Mizunaga, T. Nakashizuka, S. Natume, K. Niiyama, T. Ohkubo, S.-i. Sawada, H. Sugita, S. Takatsuki, and N. Yamanaka. 1999. Geographical variation in the early regeneration process of Siebold's Beech (*Fagus crenata* BLUME) in Japan. Plant Ecology **140**:129-138.

Ishihara, M., K. Ishida, H. Ida, A. Itoh, T. Enoki, T. Ohkubo, T. Kaneko, N. Kaneko, S. Kuramoto, T. Sakai, S. Saito, H. Sakio, M. Sakimoto, H. Shibano, H. Sugita, M. Suzuki, M. Takagi, A. Takashima, M. Takyu, N. Tashiro, N. Tanaka, N. Tokuchi, K. Namikawa, K. Niiyama, N. Nishimura, M. Noguchi, H. Nomiya, T. Hiura, A. Fujiwara, D. Hoshino, K. Homma, A. Makita, T. Masaki, T. Yoshioka, and T. Yoshida. 2010. An introduction to forest permanent plot data at Core and Subcore sites of the Forest and Grassland Survey of the Monitoring Sites 1000 Project. Japanese Journal of Ecology **60**:111-123 (in Japanese).

Larson, A. J., J. A. Lutz, R. F. Gersonde, J. F. Franklin, and F. F. Hietpas. 2008. Potential site productivity influences the rate of forest structural development. Ecological Applications **18**:899-910.

Masaki, T., M. Oguro, N. Yamashita, T. Otani, and H. Utsugi. 2017. Reforestation following harvesting of conifer plantations in Japan: Current issues from silvicultural and ecological perspectives. Reforesta **3**:125-142.

Royle, J. A., and R. M. Dorazio. 2008. Hierarchical modeling and inference in ecology: the analysis of data from populations, metapopulations and communities. Academic Press, Amsterdam.

Tarboton, D. G. 1997. A new method for the determination of flow directions and upslope areas in grid digital elevation models. Water Resources Research **33**:309-319.

Yamaura, Y., H. Oka, H. Taki, K. Ozaki, and H. Tanaka. 2012. Sustainable management of planted landscapes: lessons from Japan. Biodiversity and Conservation **21**:3107-3129.

Yokoyama, R., M. Shirasawa, and R. J. Pike. 2002. Visualizing topography by openness: a new application of image processing to digital elevation models. Photogrammetric Engineering and Remote Sensing **68**:257-266.

Zuur, A. F., A. A. Saveliev, and E. N. Ieno. 2012. Zero inflated models and generalized linear mixed models with R. Highland Statistics, Newburgh.
